# Mapping the rare disease stakeholders in India

**DOI:** 10.1371/journal.pgph.0003516

**Published:** 2026-03-26

**Authors:** Mohua Chakraborty Choudhury, Jerry Philip George, Prashanth N. Srinivas

**Affiliations:** 1 DST Center for Policy Research Indian Institute of Science, Bengaluru, India; 2 Institute of Public Health, Bengaluru, India; 3 Isaac Centre for Public Health, Indian Institute of Science, Bengaluru, India; 4 Sree Chitra Institute for Medical Sciences and Technology, Thiruvananthapuram, India; PLOS: Public Library of Science, UNITED STATES OF AMERICA

## Abstract

Rare diseases (RD) are not rare collectively, affecting around 300 million people globally and 96 million in India. These diseases have not been prioritized in most low- and middle-income countries’ health policies. India launched its first functional RD policy in 2021. Successful policy implementation requires the active participation of diverse stakeholders. In the context of rare diseases, such collaboration has been particularly instrumental in driving policy execution and systemic transformation. RDs are not well researched in India and there are no studies on mapping and analysis of RD stakeholders. Thus, this study aims to comprehensively map all stakeholders in the RD ecosystem in India, to understand their power, positions, influence, and needs. In-depth analysis of stakeholder perspective was done through semi structured interviews and news-media analysis. This is an exploratory study aimed to map all RD stakeholders and present their perspectives without drawing conclusive inferences. We found that stakeholders such as local and international patient organizations, think tanks, research communities, policymakers, local and multinational companies engage extensively with RD activities. However, high influence is limited largely to policymakers, and a few rare disease specialist physicians, with some participation of other groups. A significant lack of awareness and knowledge about RDs was found among general healthcare professionals and allied health professionals. This places a disproportionate burden on a limited pool of specialized doctors, predominantly concentrated in a few cities. Thus, for better implementation of RD policy it is crucial to encourage diverse stakeholder engagement and participation. The study highlighted stakeholders with high and low engagement. Highly engaged stakeholders should be leveraged for policy implementation, while awareness and training programs need to be targeted towards low engagement groups.

## 1. Introduction

Rare diseases (RDs) are severe debilitating conditions that affect a small portion of the population. RDs collectively affect a substantial population of around 300 million individuals worldwide [1] and pose a significant challenge to the healthcare system of any country. These diseases have a severe impact on the lives of patients and their caregivers. In India, about 96 million patients are affected by RDs [2,3]. There has been very little support from the government for RDs and they did not feature in the country’s health policy agenda until recently. However, driven by patient advocacy movements in 2017 Ministry of Health and Family Welfare, Government of India launched the country’s first National Policy for Treatment of RDs which was put in abeyance in 2018, and a revised version, National Policy for Rare Diseases (NPRD) was adopted in 2021 [3,4]. The vision of the NPRD in India is to ensure early diagnosis, affordable treatment, comprehensive support systems, and collaborative research to improve the lives of RD individuals’ policy [5]. The policy faced mixed reviews from different stakeholders. However, most appreciated it as a move in the right direction [6].

Globally changes in RD ecosystem has been driven by collaborative efforts of multiple stakeholders including patients, caregivers, healthcare providers, researchers, advocacy groups, and policymakers Stakeholders can raise awareness about RDs, facilitate early diagnosis, provide research inputs and ensure access to appropriate healthcare services [7].

Thus, to ensure effective adoption of the RD policy it is crucial to identify all the stakeholders in the RD ecosystem, and understand their position, power, role, and influence [8]. However, RD ecosystem is not very well studied in India and no studies have extensively mapped RD stakeholders in the country. A pioneering study in 2014 explored the RD community in India, mentioned a preliminary outline of possible stakeholder groups [9]. Our two other publications studied specific stakeholder groups: the patient organizations and orphan medicinal product organizations [10,11]. However, a comprehensive study mapping of all stakeholders group in the RD ecosystem has not been done yet. Therefore, realizing the gap, we attempt to identify all major stakeholder groups in India and understand their knowledge, interest, position, and power in the RD ecosystem that would impact implementation of NPRD. This study will be beneficial to map RD stakeholders in similar other settings of low- and middle-income countries as well.

## 2. Methodology

**Fig 1:**
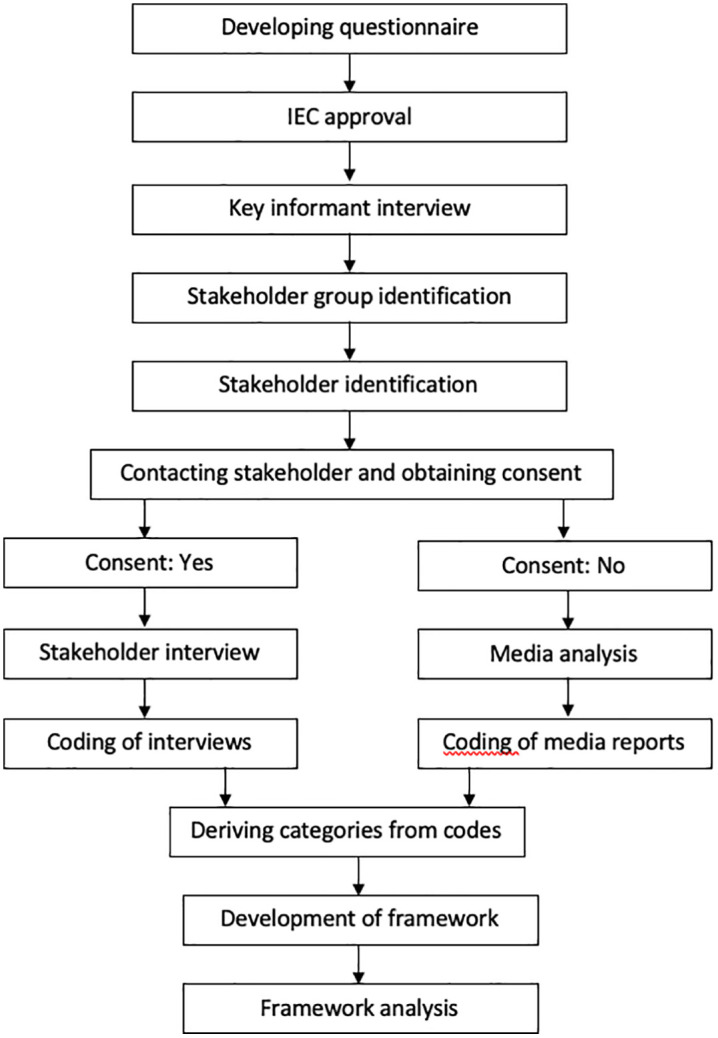
A flowchart showing the key steps of methodology.

We used qualitative research methodology that involved in-depth interviews and news-media analysis. Our exploratory mapping study aimed to identify key stakeholder groups, reaching out to as many individuals as possible within each group to achieve content saturation. Despite repeated efforts, some stakeholders did not respond to our interview invitations. To capture the perspectives and activities of these groups, we conducted a news-media analysis focused on their involvement and views related to RDs. The different steps involved in the study are laid out in Fig 1 and each step is described below:

### 2.1 Developing questionnaire

The questionnaire for a semi-structured interview used in this study was collaboratively developed by the corresponding author, MCC and PNS, a co-author and mentor in this study. Drawing upon MCC’s knowledge and research experience of the RD ecosystem over the past six years, as well as PNS’s extensive expertise in the field of public health, we designed the questionnaire to capture comprehensive insights from the stakeholders for the RD ecosystem. The semi-structured questionnaire is available in S1 File.

### 2.2 Ethics, consent, and permission

To ensure ethical conduct, the study received approval from the Institutional Ethics Committee of the Institute of Public Health for study number IEC-FR/01/2021 on 04/02/2021. The potential interviewees were provided with an information sheet containing information about the researchers’ affiliation, the source of funding, the study objectives, and the investigators’ plan to publish the findings in an academic journal without seeking their additional approval. Participants were required to respond positively to an interview invitation sent by email and sign an informed consent form to indicate their willingness to participate in the interviews. Lastly, participants were informed that they would not receive any compensation for participating in the study.

### 2.3 Data collection: semi-structured interviews

Interviews were conducted in two phases, first with four key informants, which helped to identify the stakeholders and categorize the stakeholder groups. In the second phase, 33 stakeholders were identified belonging to 10 stakeholder groups who were interviewed with the tool developed in this study. The groups, in which stakeholders did not consent to interview, data was collected through media analysis.

#### 2.3.1 Key informant interviews.

In the first interview phase, four key informants were interviewed to identify stakeholder groups, facilitate contact with the stakeholders and get insights to strengthen the interview tool. A key informant was identified as a person who understands the RD ecosystem in India and has knowledge about different stakeholder groups, but is not inherently a stakeholder. These interviews served as a valuable means of identifying and categorizing stakeholders into relevant groups. However, these interviews were not included as part of the stakeholder analysis.

#### 2.3.2 Stakeholder identification and categorization.

Stakeholder inclusion was guided by the study objective of comprehensively mapping the rare disease (RD) ecosystem in India using the WHO health systems framework. Stakeholders were identified through triangulation of key informant interviews, literature review, and the author’s prior research experience, ensuring representation across service delivery, governance, financing, research, and product development.

A total of 10 distinct stakeholder groups within the RD ecosystem was identified. The selected stakeholder groups collectively represent all core building blocks of the WHO health systems framework. Doctors (general and RD specialists) and allied health professionals align with service delivery and the health workforce, as they provide diagnosis, treatment, and long-term care. Patient organizations (Indian and international) and think tanks contribute primarily to leadership and governance through advocacy, accountability, and policy influence, while also supporting service navigation. Policymakers and government actors anchor governance and financing, shaping regulation, program design, and resource allocation. The research community supports health information systems and innovation generation, whereas multinational companies and orphan medicinal product organizations relate to access to essential medicines and technologies and financing through product development, availability, and market participation. Together, these groups ensure system-wide representation of the RD ecosystem. T. We contacted several stakeholders across these groups but were finally able to get interviews with a total of 33 stakeholders.

Data saturation was assessed iteratively within stakeholder categories and was considered achieved when consecutive interviews yielded no new themes relevant to RD care, policy, or advocacy. In some groups, further recruitment was limited by non-response despite repeated contact attempts; in these cases, saturation was determined based on thematic convergence across available interviews and consistency with findings from other stakeholder groups.

#### 2.3.3 Stakeholder interviews.

In-depth semi-structured interviews were conducted with a total of 33 eligible stakeholders who consented to participate in the study. The interviews were conducted between May 2021- July2022. Interviews were conducted via video conference and recorded with participants’ prior consent. Written and oral consent were obtained after a detailed explanation of the informed consent form (ICF). Participants were given sufficient time to review the ICF and ask questions. Oral consent was reconfirmed at the start of each interview. A sufficient number of interviews were conducted within each stakeholder group to enable data saturation. As an exploratory study, this process enabled diverse stakeholder views without aiming for conclusive inferences.

#### 2.3.4 News-media analysis.

Despite multiple invitation requests, stakeholders of some groups, such as multinational companies, policy makers, government officials, and the judiciary did not respond to our invitation, resulting in low representation of these groups in our study. As a result, an alternative data collection method, news-media analysis, was adopted. To conduct this analysis, relevant English language newspapers were searched for news articles related to RDs and the respective stakeholders. The specific keywords used the formula “#Stakeholder name”,“Rare Disease”, “India” on Google News search, covering the period from March 2017 to September 2022. Results from the first five pages of the search were considered and the articles were then subjected to qualitative analysis as a substitute for in-depth interviews.

### 2.4 Data analysis

#### 2.4.1 Qualitative analysis of stakeholder interviews.

The transcriptions of the in-depth online interviews were generated using Otter AI, and the qualitative analysis was performed using QDA Miner 6 for Windows. Initially, an iterative inductive coding approach was utilized to code at least two interviews from each stakeholder group. This coding enabled the derivation of codes and categories that were subsequently used for the deductive coding of the remaining interviews. By incorporating both inductive and deductive coding methods, a hybrid approach was utilized for the qualitative analysis. However, in some cases, new codes were added iteratively as well.

#### 2.4.2 Qualitative analysis of news-media articles.

The media analysis also involved a qualitative analysis of the relevant newspaper articles in popular Indian print media. In this approach, the entire articles were used as a substitute for in-depth interview transcripts. Any quotes or references to a stakeholder were extracted to form their respective perspective. Deductive coding was then applied to these articles, based on the codes and categories that were formed from the in-depth interview analysis. By utilizing this approach, it was possible to further explore the themes and insights related to the stakeholder groups that had limited participation in the in-depth interviews. The findings from the news-media analysis were also integrated with those from the in-depth interviews to provide a more comprehensive understanding of the RD ecosystem and the stakeholders involved.

#### 2.4.3 Framework based analysis.

To analyze the codes, the stakeholder analysis framework originally developed by Balane MA et al. [12] was adapted and customized as necessary. After thorough consideration, this framework was determined to be appropriate for the study, with only minor changes required to fully align it with the specific context of the RD ecosystem being analyzed. Within this adapted framework, stakeholders were assessed and evaluated based on four key characteristics: knowledge, interest, power, and position. Within each characteristic, specific domains were identified, each stakeholder was scored on a four-point (0–3) scale, and the sum of scores of all the domains within each characteristic was used for further inter and intra group analysis of the stakeholders.

The scores obtained were used to analyze characteristics of stakeholder groups, enabling a comprehensive understanding of their roles and perspectives. To fit the context of the study, the definition and interpretation of the value scale were also modified from the Balane et al. framework. The ‘knowledge’ and ‘interest’ characteristics in the modified framework are fairly similar to the components in the original framework. In the ‘power’ characteristic, an additional domain of ‘no power’ in the value scale interpretation was added as a few stakeholders in the study had no influence over policymaking. Similarly, in position, value scale definition and interpretation were modified so as to ascertain the position that each of the stakeholders took in the study. Table 1 gives a detailed description of the modified framework and lists the domains used in each characteristic.

**Table 1:** Modified stakeholder analysis framework for rare disease stakeholder analysis.

Stakeholder (SH) Characteristics	Characteristic definition	Domain	Value scale definition	Value scale interpretation
Knowledge	SH’s knowledge and understanding of the RDs and RD policy	• Knowledge of RDs • Knowledge of RD policies • Knowledge of RD management • Understanding of Indian RD ecosystem	0-No knowledge in the listed domains	0—No knowledge
			1-Knowledge in 1 listed domains	1—Limited knowledge 3—High Interest 0—No power 1---Low power
			2-Knowledge in 2 listed domains	2—General Knowledge
			3-Knowledge in 3 or more listed domains	3—Extensive knowledge
Interest	SH’s interest in RD policy development, implementation and contribution to RD ecosystem	• Interest in RD policy development • Interest in RD policy implementation • Contribution to RD ecosystem	0-No interest in the listed domains	0—No Interest
			1-Interest in atleast 1 domain	1—Limited Interest
			2-Interest in 2 listed domains	2—General Interest
			3-Interest in 3 listed domains	
Power	The ability of the SH to influence RD policy development and implementation	• Political authority • Financial authority • Technical (Domain) expertise • Leadership	0-Stakeholder possesses and has no control over any of the listed domains	
			1-Stakeholder possesses and has control over use of one to two domains of power, low potential to affect policy implementation	
			2-Stakeholder possesses and has control over use of two to three domains of power, has moderate potential to affect policy implementation	2—Medium power
			3-Stakeholder possesses and has control over use of three to four domains of power, has high potential to affect policy implementation	3—High power
Position	Whether the stakeholdersupports, opposes or is neutral about policy implementation	A. degree of support or opposition to policy expressed through use of potential power (sources of power) 1. Neutral 2. Supporter 3. Moderate Supporter 4. Opponent B. Actions taken to demonstrate support or opposition to policy 1. No actions taken 2. Actions taken	0- Opponent: A4 and B1 or B2	0---Opponent: Stakeholder uses potential power to moderately acting against policy implementation
			1- Neutral: A1 and B1	1—Neutral: Stakeholder does not use potential power and does not act for or against policy implementation
			2- Moderate Supporter A3 and B1 or B2	2—Moderate Supporter: Stakeholder uses potential power to moderately act in support of policy implementation
			3- Supporter: A2 and B1or B2	3—Supporter: Stakeholder uses potential power to strongly act in support of policy implementation.

The original framework was adopted from Bayne et. al. was modified to suit the rare disease ecosystem in India.

Through the scoring, we intended to capture the breadth of the characteristics of each stakeholder across all the listed domains. A stakeholder having a score in more than one domain is rated higher on the value scale. If a stakeholder’s score lies only in one domain, it will be rated lower even if he/she has deep expertise in that domain. Thus, the matrix does not aim to measure the depth of stakeholders in each domain because the focus of the matrix is to elucidate the overall understanding of each stakeholder about the RD ecosystem and not depth of knowledge in their respective domain. Also, the authors do not have expertise to assess depth of knowledge in each domain and the questionnaire was designed accordingly.

## 3. Results

A total of 33 semi structured interviews were conducted with stakeholders. Additionally, news-media analysis method was used to get insights of those stakeholder groups who did not respond to interview invitations.

### 3.1 Key informant interviews

Four key informants were interviewed - a public health practitioner and researcher working with RD patient group for last 15 years who facilitated RD stakeholder interactions for the government prior to the formulation of the RD policy, a senior Indian Council of Medical Research official with experience overseeing RD initiatives and another one is a scientist and patient advocate. Through the key informant interviews we were able to identify and map many stakeholders in the country and group them into 10 stakeholder groups.

### 3.2 Profile of stakeholders

A list of all potential stakeholders was created with information about their affiliations, and each stakeholder was contacted. Many participants spoke from personal experience rather than on behalf of their organizations. However, their viewpoints might be seen as a reflection of their experience and engagement with the respective organizations. A total of 33 in-depth interviews were conducted across various participant categories. Stakeholder groups with insufficient representation, due to non-response despite multiple attempts, were studied through news-media analysis. A summary of the stakeholder groups and method of data collection used is given in Table 2.

**Table 2:** Table listing the groups of stakeholders, acronym, and data collection methods.

Sl. No.	Stakeholder group	Acronym	Method of data collection
1	Doctors (non-RD specialists)	NRDS	In-depth interviews
2	Doctors (RD specialists)	RDS	In-depth interviews
3	International patient organizations	INTO	In-depth interviews
4	Indian Orphan Medicinal Product Organization	OMPO	In-depth interviews
5	Indian patient organizations	PATO	In-depth interviews
6	Think tanks	TTNK	In-depth interviews
7	Research community	RCOM	In-depth interviews
8	Multinational companies	MNCO	In-depth interviews & news-media analysis
9	Policymakers and government	PMKR	In-depth interviews & news-media analysis
10	Allied health professionals	AHPR	In-depth interviews

This table provides an overview of the stakeholder groups included in the study, along with their corresponding abbreviated codes and methods of data collection. (For ease of communication, these acronyms are used throughout the manuscript and figures).

### 3.3 Data analysis

**Fig 2:**
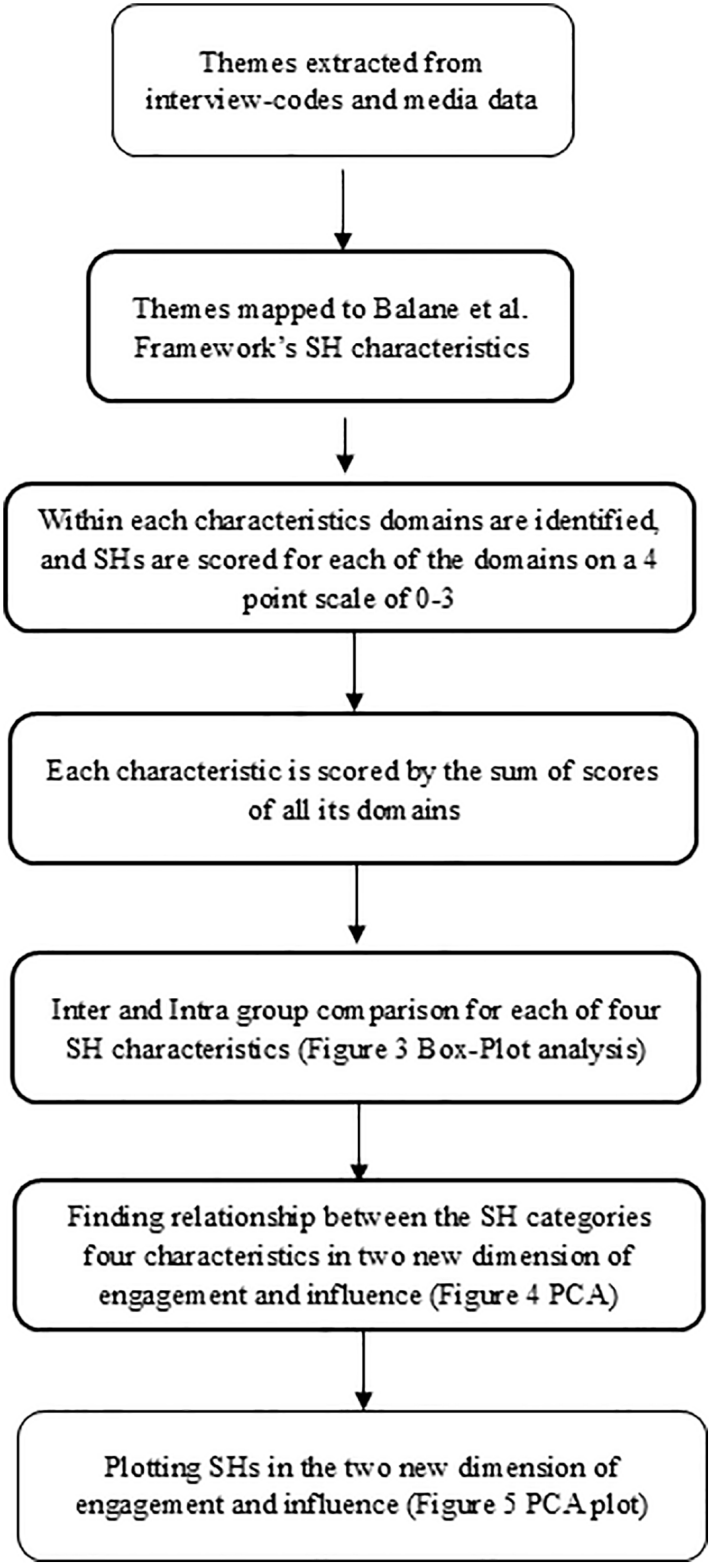
A flowchart summarizing the steps of analysis of the stakeholder interview and media data.

Fig 2 summarizes the different steps of data analysis, which is described in this section:

#### 3.3.1 Interview data analysis.

Inductive coding was used to analyze the transcript of the interviews and derive themes. The derived codes were grouped into seven major themes described here.

(i) **Experience**: This encompassed the personal and professional experience the stakeholders had in the RD ecosystem. It also included codes on contributions made towards advocacy, research, and activities related to RD.
(ii) **Perspectives and Knowledge:** This includes codes on knowledge of RDs, RD policies, diagnosis, treatment, clinical trials, the registry, and orphan drugs. It also included codes for understanding the Indian RD ecosystem and the drivers of change.
(iii) **Challenges**: The RD stakeholders faced challenges at multiple levels, including sociocultural, policy-level, research, infrastructure, healthcare access, treatment cost, inclusivity in policymaking, insurance, and healthcare worker competency. These were essentially captured into codes and helped in forming the category of challenges in the RD ecosystem in India.
(iv) **Recommendations:** Similarly, recommendations included codes on policy refining, social and emotional support, awareness improvement, funding, screening, and improving access to diagnosis and treatment.
(v) **Influence:** Influence on RD-related policies had codes classified into two categories: direct influence and indirect influence. Direct influence occurs when a stakeholder has a direct role in policymaking, whereas indirect influence occurs when a stakeholder exerts influence through other activities such as empowering PAGs or holding meetings, discussions, or conferences to foster a favorable policy environment.
(vi) **Alliance:** This theme identified the networks that existed between various stakeholders was alliance. Codes in this category included the alliances mentioned by the stakeholders such as those between industry, academia, the PAG, the government, and international organizations.
(vii) **Implementation:** In the implementation category interest, attitudes, challenges, and recommendations pertaining to the NPRD were captured.

#### 3.3.2 News-media analysis.

News-Media analysis helped to compensate for under representation in some stakeholder groups mainly in policymakers and government, and multinational companies. A total of 127 articles were extracted and reviewed from the timeline of 2017–2020 (listed in Table 3 and detailed in S2 File). Yearly distribution of newspaper reports covering news and stories related to RDs was low, which showed the lack of media interest in this field. However, the coverage has shown some increase over time with the release of the first National RD Policy in 2017. All stakeholder included belonged to the Policymakers (PMKR) groups. We searched mainly for central government organizations such as Ministry of Health and Family welfare (MOHFW), Ministry of finance (MOF), Niti Aayog, Central Drugs Standard Control Organization (CDSCO) and Drugs Controller General of India (DCGI). Two state governments Karnataka and Kerela showed highest number of articles related to initiatives for RDs compared to other states in India. MOHFW showed the highest number of hits followed by Niti Ayog. Articles featuring government initiatives in Kerela ranked next. Judiciary has been seriously involved through litigations and cases filed by parents which are rightly reflected in our data.

**Table 3:** Number of articles found through news-media analysis for each stakeholder.

Key word	Number of relevant articles						
	2017	2018	2019	2020	2021	2022	Total
Ministry of Health and Family welfare, Govt of India (MOHFW)	3	1	2	6	4	5	21
Niti Aayog	2	0	2	5	4	5	18
Ministry of Finance, govt of India (MOF)	6	3	2	1	3	4	19
CDSCO	1	1	2	1	3	4	12
DCGI	0	2	0	3	3	3	11
Court	2	4	2	4	3	4	19
Karnataka	2	1	0	4	3	4	14
Kerala	3	1	0	4	3	3	14

This table presents the number of articles for each stakeholder through news-media analysis. The number of articles reviewed depended on the availability and relevance of articles related to each keyword.

Relevant quotes and information related to each of the above-mentioned characteristics for stakeholders in this group were captured from news articles and analyzed along with interview-data.

However, the news-media analysis did not reveal details about the activities and involvement of each of these stakeholders in RD ecosystem therefore we clubbed findings from all the government agencies under single stakeholder category of policymaker.

### 3.4 Framework based analysis: mapping themes derived from interview and news-media analysis data to Balane et al stakeholder framework

The extracted themes were then mapped to the Balane MA et al framework which has 4 key stakeholder characteristics: knowledge, interest, power, and position. These characteristics were adopted and defined based on the context of the RD ecosystem in India. Knowledge refers to the stakeholder’s knowledge and understanding regarding RDs and their ecosystem in India. Interest refers to stakeholders’ interest in RD policy development, implementation, and contribution to the RD ecosystem. Power refers to the ability of the stakeholder to influence RD policy development and implementation. Position refers to the alignment of the stakeholders whether they support, oppose, or are neutral about policy implementation. The domains of each of the 4 characteristics are enlisted in Table 4.

**Table 4:** Themes from interview and news-media analysis.

Sl. No.	Themes	Description of the theme	Framework characteristic
1	Experience with RD	Included codes on personal and professional experience with RD and contributions made in advocacy, research and activities related to RD	Interest, Knowledge
2	Knowledge on RDs and related policies	Included codes on knowledge of RDs, RD policies, diagnosis, treatment, clinical trials, registry and orphan drugs. It also includes codes on understanding of the Indian RD ecosystem and the drivers of change.	Knowledge
3	Challenges in RD ecosystem in India	Includes codes on challenges at various levels – sociocultural, policies, research, infrastructure, healthcare access, cost of treatment, inclusivity in policymaking, insurance and competency of health care workers	Knowledge
4	Recommendations	Included codes on policy refining, social and emotional support, awareness improvement, funding, screening, improving access to diagnosis and treatment	Knowledge, Interest
5	Influence on RD related policies	Codes are divided into 2; direct and indirect influence. Direct influence is when a stakeholder has direct role in policymaking, and indirect influence represents influence via other activities such as empowering PAGs, meetings/discussions/conferences held to make favourable environment for policy. How they	Power
6	Alliance	Includes codes about knowledge on alliance between policies and on alliance between organizations such as alliance between industry, academia, PAG, govt and international organizations	Power, Knowledge
7	Implementation	Includes codes on role in implementation, interest, attitude, challenges and recommendations pertaining to NPRD	Interest, Power, Position

This table presents themes derived from the qualitative analysis of in-depth interviews and news-media analysis mapped to the stakeholder characteristics of Balane MA et al, displayed in the right column.

The summary of themes and codes is given in Table 4 and detailed codes are given in S3 File.

### 3.5 Framework-based scoring of data

**Fig 3:**
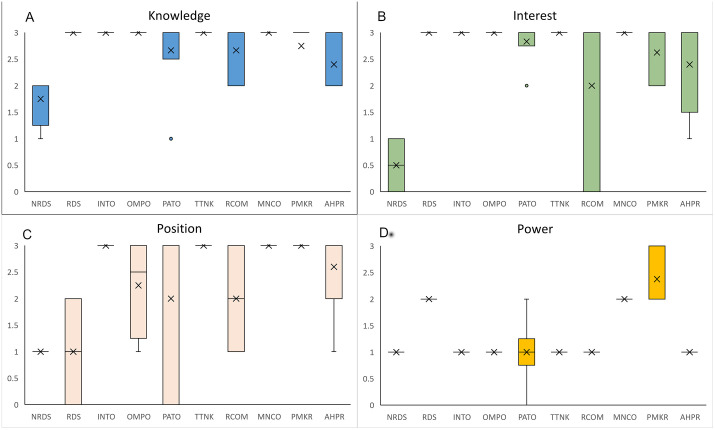
Box and Whisker Plots illustrating the distribution of scores of individual stakeholders within each stakeholder category for the four stakeholder characteristics: 3A knowledge, 3B interest, 3C position, and 3D power. The whiskers extend to the minimum and maximum values, excluding outliers represented as individual data points beyond the whiskers. The cross marks indicate mean scores obtained by each stakeholder category.

Overall views of inter and intra group variation between the stakeholder groups across four characteristics: knowledge, interest, position and power are illustrated in Fig 3 showing a Box and Whisker plot. The shape of the box plot shows how the data is distributed within each group. The cross mark and bold line show the mean and median score of the group. Below for each characteristic the distribution of stakeholder scores is described, the mean scores are mentioned in the description. The detailed score matrix is given in S4 File and a example scoring chart is given in S5 File.

#### 3.5.1 Knowledge.

Knowledge was assessed from 4 key domains namely knowledge of RDs, knowledge of RD policies, knowledge of RD management, and understanding of the Indian RD ecosystem which were derived from the codes of interviews.

Fig 3A shows extensive knowledge reflected by 3.0 score in RDS, INTO, OMPO, TTNK, and MNCO, followed by PMKR (2.7), and PATO (2.6), RCOM (2.6). AHPR (2.4), and NRDS (1.75) exhibit significant intra group variation, indicating a wide range of knowledge scores within these groups. A limited/general knowledge was observed among NRDS (1.8), these are practicing physicians who are not specialized in RDs. Most reported they have not encountered an RD patient and are not aware of various diagnostic possibilities.

Stakeholders who possess limited or general knowledge in one domain may have extensive knowledge in other domains. RDS has extensive knowledge of disease, diagnosis, treatment, and care. However, all of them might not be well acquainted with RD policies. PATOs actively engage in various activities such as campaigning, fund generation, and mobilizing various actors to gain a collective benefit. Many of the actors in these organizations are patients or their caretakers, who have lived experiences of the disease and its burden. Years of advocacy, campaigning, and legal actions by some PATOs eventually compelled the judiciary to intervene, directing the government to formulate a new policy. This led to the creation of the National Rare Disease Policy in 2017 and revision in 2021 [13]. Of the five PATOs in the study, four showed a high score of 3 in knowledge only one showed a score of one which reduced the average score of knowledge in PATO group. This stakeholder scored low on RD policy knowledge as the disease specific group functions independently and is not associated with other RD groups.

INTOs offer support in many roles, such as providing technical expertise, introducing to the policies and interventions used in other countries, mobilizing funds, and helping to represent India on various international platforms pertaining to RDs.

OMPO, RCOM, MNCO, and TTNK possess extensive technical knowledge in their own respective areas of interest. RCOM contributes to development of new therapies, diagnostics and generates knowledge about diseases. TTNK helps in organizing existing evidence to support policy making. MNCO and OMPO are commercial players who have their own research wings that aid in creating new products that are affordable to patients in India. For instance, one OMPO specializes in nutritive products for RD patients, focused on developing affordable, India-specific alternatives to previously imported, costly products. NRDS and APHR have limited knowledge about the clinical aspects of RDs including screening and diagnosis. Their experience with RDs is low both in terms of academic exposure to RDs and seeing RD patients. AHPRs included a speech therapist, a physiotherapist, a public health professional and two genetic counsellors. All of them were associated with RD patient groups and have provided service to RD patients. Among them the genetic counsellors showed the highest score of knowledge. A few NRDS at primary health centers revealed that they have never come across any patients during their practice.

#### 3.5.2 Interest.

Interest was assessed based on 3 domains, namely, interest in RD policy development, interest in RD policy implementation, and contribution to the RD ecosystem. As seen in Fig 3B, high interest of 3.0 score was observed among RDS, INTO, OMPO, TTNK, MNCO, RDS and PATO. An average general/limited interest was observed in PMKR (2.6), AHPR (2.4) and RCOM (2) whereas low interest in NRDS (0.5).

RCOM exhibits significant intra group variation, followed by AHPR, NRDS, and PMKR, indicating a wide range of interest scores within these groups. Conversely, most stakeholders in the remaining groups demonstrate a similar high score in interest, suggesting a more consistent distribution within these groups.

TTNK and RCOM are more inclined towards the academic aspects and therefore have an interest in the policy recommendations which can facilitate research activities in the ecosystem. Conversely, the interest of multinational companies in RD policymaking may, by their interest of making their drugs accessible to Indian patients. PMKRs exhibited a varied range of interest with, scores ranging from 2 to 3. A limited interest was shown by a few stakeholders in this group as competing priorities in a resource-constrained setup limit their ability to address the pertinent issues related to RDs significantly.

#### 3.5.3 Position.

In Fig 3C, INTO, MNCO, PMKR and TTNK shows uniform and high mean score of 3.0 demonstrating strong support for the policy. The plot reveals wide intragroup variation in the OMPO (1.0), PATO (2.0), RCOM (2.0), and RDS (2.3) groups, suggesting a diverse range of positions among stakeholders within these groups. On the other hand, the NRDS (1.0) group is characterized by a neutral position, indicating a lack of clear alignment with any specific stance.

Position was assessed based on 2 key domains: the degree of support or opposition to policy expressed through the use of potential power and actions taken to demonstrate support or opposition to the policy. Position refers to the alignment of the stakeholder whether they support, oppose, or are neutral about policy implementation. NRDS took a neutral position as they had a limited understanding of the RD ecosystem or the activities pertaining to the same. A few stakeholders in PATO and one member of RDS opposed the policy owing to a few limitations of the policy. PATO and RDS have an extensive first-hand understanding of RDs and have been proactive in this space. They understand the limitations of the policy and how policies around the world pertaining to RDs are functioning.

#### 3.5.4 Power.

As seen in Fig 3D uniform low power score of (1.0) is seen among all members in seven of the stakeholder groups viz NRDS, INTO, TTNK, RCOM, AHPR. Notably, the PMKR stands out with highest mean score (2.4) and wide intragroup variation. Followed by MNCO and RDS groups demonstrating medium power levels of 2.0.

Power was also assessed based on 4 domains, namely, political authority, financial authority, technical (domain) expertise, and leadership. Power was concentrated more in PMKR followed by MNCO and RDS. PMKRs demonstrated dominance in all 4 domains. MNCO held a moderate level of power. On the other hand, RDS had a medium power to influence policy making and implementation, as most of them were actively involved in the policymaking process through participation in various task forces.

### 3.6 Inter-relationship of the four characteristics: new dimension of engagement and influence

**Fig 4:**
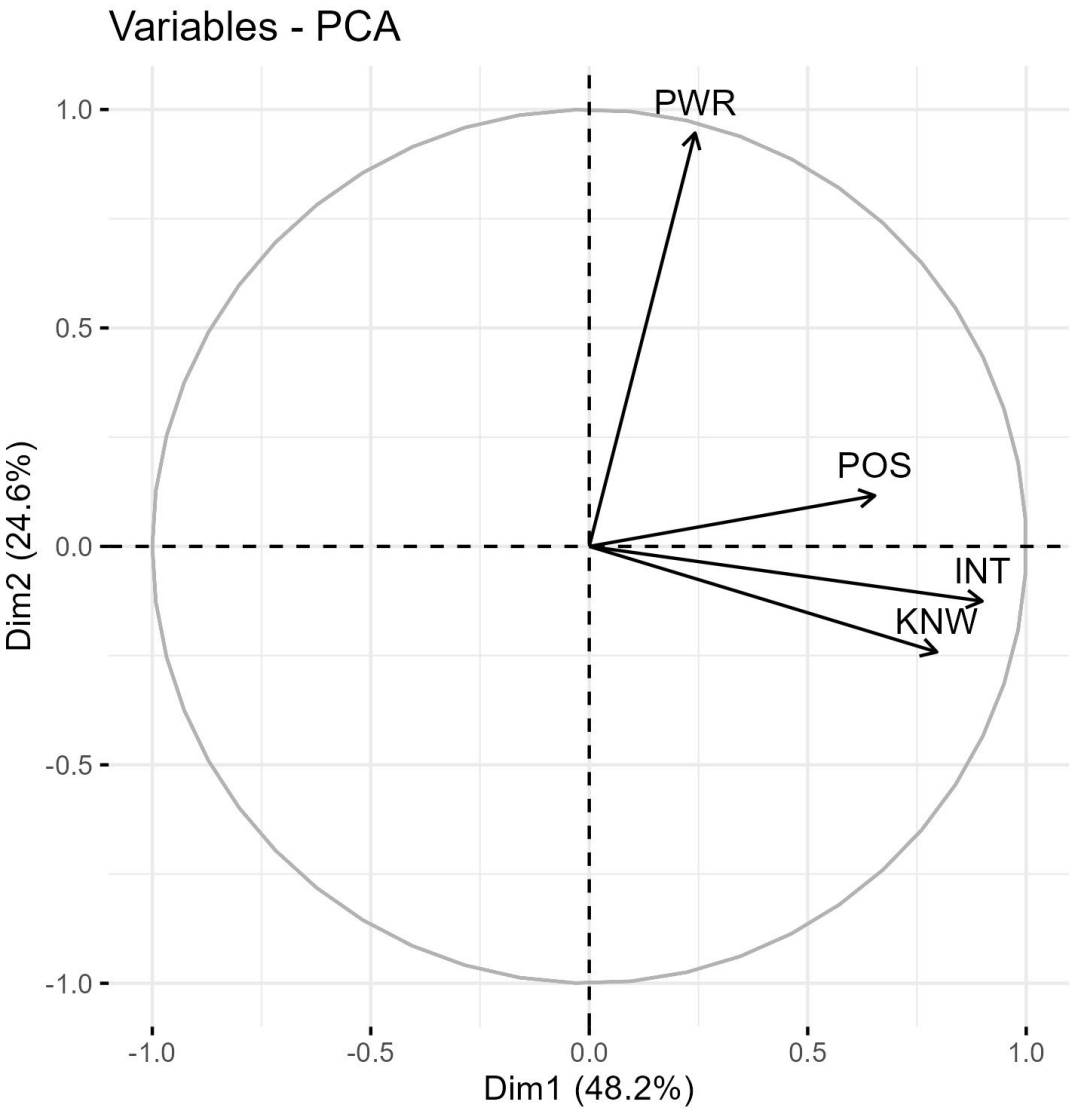
Principal Component Analysis (PCA) of the Four Characteristics. Influence (Y-axis) vs. Engagement (X-axis) The presented figure illustrates the results of a Principal Component Analysis (PCA) performed on the four characteristics: knowledge, interest, power, and position. The analysis generated two new components: Influence (represented on the Y-axis) and Engagement (represented on the X-axis). The PCA allows for the reduction of the multidimensional data into two principal components that capture the majority of the variance in the original dataset. The positioning of data points on the scatter plot indicates the level of influence (vertical axis) and engagement (horizontal axis) exhibited by each stakeholder. This representation enables a visual understanding of the relationships and patterns between the stakeholder characteristics and their impact on influence and engagement within the studied context.

A principal component analysis was performed to investigate the inter-relationships among the four characteristics observed in the stakeholders. In Fig 4, the X and Y axes represent composite scores derived from the four characteristics. The angles formed by the three components (knowledge, interest, and position) are more acute, indicating a strong association among these variables. These three components are primarily aligned with the X-axis and a new composite dimension termed ‘engagement’ is created by combining them. Similarly, the dimension of ‘influence’ is derived from the power variable, which is predominantly aligned with the Y-axis.

**Fig 5:**
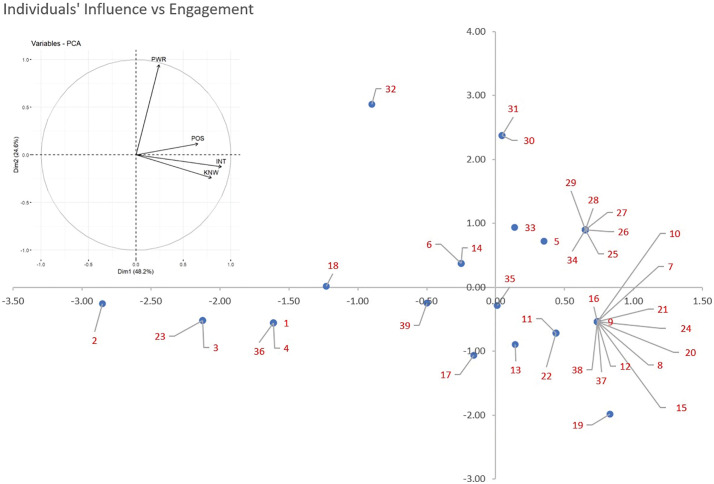
Principal component analysis and scatter plot of all the 39 stakeholders belonging to different stakeholder groups in the study. The scatter plot visually represents the positioning of each stakeholder in the reduced dimensional space derived from the PCA. The X and Y axes correspond to the influence and engagement respectively obtained from the analysis. The scatter plot provides insights into the clustering, dispersion, and relationships among the stakeholders.

Using X Axis as Dimension 1 of Engagement and Y axis and dimension 2 influence each stakeholder’s original scores on the four characteristics are transformed into two principal component scores and plotted on the two axes shown in Fig 5.

The scatter plot reveals that 26 out of 39 data points are concentrated on the right side of the Y-axis, indicating high positive engagement in RD-related activities among stakeholders. However, most points (26) fall below the X-axis, highlighting that only few exhibits significant influence on policymaking.

In the first quadrant, showing high engagement high influence a cluster of six stakeholders (25,26,27,28, 29,34) representing PMKR and MNCO lies in the right most side with high influence and relatively high engagement. Highest influence is shown by 31, 30 which also represents PMKR groups however their engagement is close to 0. Similarly, stakeholder 33 another PMKR shows high influence but engagement close to 0. The only stakeholder (5) outside PMKR and MNCO in this quadrant is an RDS who is an important member in the RD Committee of the government.

In the second quadrant, reflecting high influence and low engagement, only three stakeholders are there. Stakeholder 32 is a PMKR showing the highest influence amongst all and low engagement. Stakeholder 6 and 14, representing RDS and PATO respectively shows some influence and low engagement. They demonstrated strong knowledge and interest but expressed opposition to the current policy, citing its inadequacy in addressing key issues, resulting in a low engagement score.

In the third quadrant: low influence, low engagement – we find nine stakeholders, all the NRDS, one RCOM, two AHPRS and one PATO from the study group.

In the fourth quadrant: low influence, high engagement – we find maximum stakeholders. A cluster of 12 stakeholders represent the highest engagement and low influence, it is comprised of all three INTOs, two stakeholders each from OMPO, TTNK, PATO, AHPR and one RCOM. This cluster is followed by a OMPO (11) and a RCOM (22) showing moderate engagement and low influence, followed by another OMPO (13) and a AHPR (35) on the axis.

### 3.7 Challenges

The interviewee’s were also asked to identify diverse range of challenges which are summarized in Table 5.

**Table 5:** Challenges in the RD ecosystem identified by different stakeholders.

Challenges	Stakeholders who identified the challenges	Specific challenges
Barriers to access to treatment	OMPO, PATO, MNCO, TTNK.	Insufficient availability and accessibility of treatments for RDs within India’s healthcare system. Limited options for patients resulting in delayed or inadequate care.
Regulatory challenges	PATO, TTNK, APHR, OMPO, MNCO	Challenges in obtaining timely access to treatments that have been approved by the U.S. Food and Drug Administration (FDA). Lengthy regulatory processes and delays in approval and availability of treatments in the Indian market.
Lack of funding	PATO, OMPO, TTNK, INTO, RCOM	Limited financial support provided by the government for RD treatments. Insufficient allocation of resources for research, development, and access to therapies.
Lack of awareness and knowledge	NRDS, PATO, INTO, TTNK	Limited awareness, knowledge and understanding of RDs among the general public and healthcare professionals. Lack of knowledge leads to delays in diagnosis, misdiagnosis, referral, and appropriate management due to low awareness.
Stigma	PATO, RDS	Reluctance among the public to acknowledge carrier status for RDs. Challenges in implementing carrier testing and counseling programs.

Most stakeholders identified a lack of treatment for RDs in India, which they attribute to the healthcare system’s inadequate accessibility and availability of care. The situation is made worse by the government’s limited funding, delays in accessing approved treatments, and low awareness among the public and physicians, which further hinder effective management. Remote areas lack expertise, while general doctors show low interest and exposure to RDs. Inadequate research, public unwillingness to accept carrier status, stigma about disease, lack of communication about policies, and gaps in healthcare coverage are other identified challenges.

### 3.8 Recommendations

Further, the interviewees were asked to list recommendations to improve the RD ecosystem based on their engagement. Their recommendations were rooted in a combination of theoretical knowledge and practical insights gained from navigating the complex landscape of RDs and are listed in Table 6.

**Table 6:** Recommendations provided by different stakeholders.

	Recommendations	Stakeholder category
1	Political will and funding crucial in fast tracking RD policy activities	PATO, OMPO, TTNK, RDS.
2	Improving accessibility of patients	PATO, TTNK
3	Improving access to clinical trials	MNCO, PATO, INTO
4	Giving importance to diagnosis irrespective of availability of treatment	MNCO, PATO, RDS, RCOM
5	Creating specialty courses or training to health professionals	PATO, TTNK, APHR
6	Improve awareness regarding RDs	PATO, INTO, NRDS
7	Creating infrastructure in the form of treatment centers as well as the disability friendly environment	PATO, PMKR
8	Creating collaborations with private and government entities	MNCO, OMPO, RCOM
9	Inclusive environment for PAGs into the policymaking system	PATO
10	Focus on RD research	RCOM, PATO
11	Prioritizing prenatal and newborn screening	PMKR, NRDS
12	Emotional and non-scientific support to be provided for patients and caretakers	PATO
13	Requirement for sensitization campaigns to promote empathy, acceptance, and support, for both visible and invisible disabilities	PATO, RDS

## 4. Discussion

Overall, the study shows that while many stakeholders demonstrate strong engagement in RD-related activities, relatively few exert substantial influence on policymaking. This is seen in the distribution of data points in the scatter plot Fig 5. Overall, some PMKR, MNCOs and a RDS show the highest influence. Whereas NRDS and AHPR show lowest influence and engagement. Other stakeholders PATO, INTO, OMPO, TTNK and RCOM also show high engagement but low influence. Although the NPRD states its development involved stakeholder consultations and expert committee recommendations [3], most stakeholders, other than the PMKR, and RDS, in our study reported no or limited direct interaction with the policymaking body. Those who submitted recommendations felt their input was not reflected in the final policy.

Although the study reflects important trends among stakeholders, it is critical to discuss the underlying data (in this case the representative stakeholders) that might have influenced the outcome and caution the generalizability of the results and also to understand the stakeholder’s perspective in the real world setting. For example: the high engagement observed among PMKRs may partly result from data bias in news-media analysis and sampling bias in selection of participants.

New media often emphasizes only the achievements of policymakers and multinational companies, while the unaddressed gaps, inactions and failures are often not covered. Additionally, the score represented collective score of the policymaker group comprising multiple government agencies such as Ministry of Health and Family Welfare (MOHFW), Indian Council of Medical Research (ICMR), CDSCO, NITI Aayog and two of the state governments from Karnataka and Kerela. Due to limited representation and insufficient media data from these entities, analysis was not feasible individually for each of the organizations. Thus, high engagement reflects the collective efforts of these entities.

Further, sampling bias was unavoidable as despite efforts to engage various government stakeholders, the response rate was low and we could interview only three representatives, all clinicians in important administrative roles.All three were directly involved in RD policy development process and provided high scores across assessed characteristics. However, these findings may not be generalized to other government officials overseeing health programs who might not have as much exposure to the RD policy development. Among the policymakers, the MoHFW showed the highest engagement, followed by NITI Aayog. Government initiatives in Kerala ranked next, while significant judiciary involvement was evident through litigations and cases filed by parents, as reflected in the news-media analysis data.

Similarly, for MNCOs, we received a response from only one SH, who has been involved with RD-related activities for a significant period and showed a high score on engagement and influence. The exorbitant cost of therapies, like the 750,000 USD per year, SMA drug, has prompted companies to engage with government and other stakeholders to explore access pathways, such as compassionate use programs [14]. These extensive interactions, might have led to higher scores for MNCOs on engagement and influence.

Furthermore, among the INTOs two were led by India- origin founders who are keen towards bridging RD initiatives across India and globally. They are heavily engaged with other stakeholders and also have had interactions with representatives from government agencies. Thus, INTO showed high engagement, which might not be the case for other global organizations.

Most diversity in scores is seen in PATO groups, with two showing high engagement, none with high influence, and few on the lower side of engagement and influence. Those on the lower side of engagement and influence are the more recently established disease-specific-groups which have been functioning more as a patient support groups. In our previous publication of analysis of patient groups in India [10], we found that different patient groups are in different stages of functioning, and only a few have evolved to be in a position to engage actively with the government. Globally, PATOs have proven to be important drivers of RD policies. Organizations such as NORD and EURORDIS are actively involved in their government RD policy design and implementation in the US and Europe, respectively [15]. RD International, a global group, has been hugely successful in pushing for the first UN Resolution of RDs in 2021 and s World Health Assembly resolution on RDs [16] [17]. PATOs in India as well have been a major force driving the government’s attention towards RDs and adoption of the NPTRD [10]. Our results show strong engagement from the few umbrella organizations and disease-specific groups. For an effective implementation of the RD policy, wider engagement with different RD-specific groups is crucial. Therefore, the empowerment of PATOs through training and access to resources and formalizing their participation in the government decision-making process is crucial.

NRDS and AHPRs showed the lowest scores in both engagement and influence amongst all the stakeholders. This is seen in other studies as well, where most healthcare workers in India lack awareness about RDs [18]. As a result, the responsibility for RD management falls on a limited number of specialists concentrated in a few major cities, which is vastly insufficient to meet the needs of the widespread and diverse RD population. Policies should therefore aim at gearing up awareness and increasing the pool of trained healthcare professionals who can care for RD patients. Further, a strong reference network system is crucial that efficiently enable these professionals to identify patients and refer them to the 12 Center of Excellence of RDs, which have been identified by the government.

OMPO, and RCOM can be important players in driving innovation in RD treatment and management and make accessible treatments available to patients [11]. However, they exhibit low power, and more concerning is that MNCOs show higher scores on power than most stakeholders. In the long run, this may widen inequity for access to treatment. Thus, OMPOs need to be empowered to ensure a sustainable RD management and treatment ecosystem. We can take examples and inspiration towards steering the indigenous orphan drug approval process from countries like Brazil which approved 21 orphan drugs and 31 clinical trials in 2019 [19]. Moreover, the Resolution of the Collegiate Board (RDC), Brazil, effective from June 1, 2020, established a regulatory framework for advanced therapy products, streamlining clinical trials and registration of innovative cell- and gene-based therapies [19]. This, along with other policies, has laid a strong foundation for RD management in Brazil, although lack of normative enforcement and insufficient funding has resulted in poor implementation [20]. India also made progress with the release of the “National Guidelines for Gene Therapy Product Development and Clinical Trials” in 2019, by ICMR and the Department of Biotechnology [21]. This paved the way for significant breakthroughs, including NexCAR19, the country’s first homegrown and globally most affordable CAR-T cell therapy for cancer [22]. TTNK is another critical stakeholder who shows high engagement and low influence. The two TTNKs worked as technical partners with the government for assessing information for the RD policy.

PATO, RCOM, OMPO, INTO, and TTNK, although have high engagement, have low influence. It is important to include this group in the decision-making process. Global consensus underscores that broad stakeholder involvement is critical to garner political support and implement RD-related policies and programs [23, 24]. Stakeholders with high engagement should be strategically utilized in policy implementation to leverage their expertise, networks, and active involvement in the RD ecosystem. They can play key roles in advocacy, creating awareness, facilitating collaborations, and ensuring the efficient execution of policy objectives.

Notably, across different groups, many of the stakeholders’ activities were directly or indirectly related to genetics and genomics, such as one of the RD specialists being a clinical geneticist, in AHPR, there were two genetic counsellors, among the researchers, all three work in genomics, two of the four Indian companies and the multinational pharma companies work in the domain of gene and cell therapy products. All of them were positioned in the second quadrant and showed high engagement. This highlights the real-world scenario where 80% of RDs have a genetic origin, and thus clinical genetics and genomics play an important role in RD diagnosis and treatment [25].

PATOs and AHPR occupy peripheral roles in decision-making within the rare disease (RD) ecosystem, despite their central role in long-term care, rehabilitation, and lived-experience knowledge. RD policy remain physician-centric, although outcomes depend heavily on professionals such as physiotherapists, speech therapists, and genetic counsellors. Their inclusion in guideline development, care pathway design, and reimbursement frameworks would better align policy with care realities. Addressing the imbalance in power requires structural rather than symbolic inclusion. Formal representation should be institutionalized in national and state RD advisory bodies, technical committees, and program review platforms, with PATOs and AHP representatives given defined consultative or decision-making roles. Dedicated funding, policy engagement training, and access to data platforms would enable more evidence-informed participation. Greater integration of AHPs into service delivery policy is needed. Co-production approaches should involve patients and caregivers early in program design improving the relevance of policies, treatment access models, and social support schemes.

## 5. Conclusion

The study offers a first-of-its-kind mapping of the Indian rare disease stakeholder perspectives. This will provide guidance for effective stakeholder engagement for policy implementation and the development of a robust RD ecosystem in the country. The study shows that policy decisions are largely influenced only by a few stakeholders: PMKR, MNCO, and RDS. However, many important stakeholders, such as PATO, RCOM, OMPO, INTO and TTNK have high potential to positively shape the RD agenda in the country based on their wide domain expertise. This highlights the need for capacity building of different stakeholders and a formal platform for inclusive participation of a wider network of stakeholders who will add value in policy implementation. Simultaneously, targeted awareness and training programs should be designed for stakeholders with low engagement such as NRDS and AHPRS to build their capacity, enhance their understanding of RD challenges, and encourage their active participation. This dual approach would ensure a more inclusive and effective stakeholder involvement, enabling a comprehensive effort to address the complexities of RDs.

## 6. Limitations of the study

- It was not possible to get adequate representation from all stakeholder groups due to lack of response, which is compensated to some extent by including news-media analysis for those stakeholder groups. However, the use of media sources to supplement stakeholder data has inherent limitations. News articles may reflect editorial framing, selective reporting, or emphasis on more visible or vocal actors, potentially underrepresenting less media-engaged stakeholders. Statements reported in the media may not capture the full nuance of stakeholder positions and cannot be probed or clarified as in interviews. To mitigate these limitations, media-derived information was used primarily for contextual understanding and was triangulated with interview data and documentary sources wherever feasible.
- Stakeholder views vary within each category, and limited representation prevents generalizability of total scores to all stakeholders in the group.
- As participation may have been more likely among particularly active or engaged individuals, the views captured may not fully represent the broader range of attitudes and knowledge levels within each stakeholder group, and findings should be interpreted with caution when considering wider generalization.
- Depth of stakeholders’ knowledge and engagement in each domain is not measured in the present study; rather focus was on overall understanding of the RD ecosystem.
- Although data collection was completed in 2022, the rare disease policy and stakeholder landscape in India has remained largely in a planning and early implementation phase across states. [26].At the same time, growing global policy momentum, including the recent WHO resolution on rare diseases, underscores the increasing relevance of understanding stakeholder structures and influence. In this context, the present mapping provides a timely baseline to inform emerging state-level implementation efforts and future system strengthening.
- Although, all interview transcripts were coded it is not possible to provide the data upon request as it may reveal sensitive information about the stakeholders. However, some masked data can be shared upon request.

## Reflexivity statement

MCC, as a RD policy researcher with six years of experience studying the RD ecosystem in India, brings extensive knowledge and familiarity with some of the stakeholders interviewed. This prior interaction may have shaped the understanding of their perspectives compared to stakeholders interviewed for the first time. PNS, with a background in public health unrelated to RDs, provided a neutral and unbiased perspective to the research. PNS intervened when any biases from MCC were noted, ensuring a balanced approach. JPG, also lacking previous experience working with RDs, offered an unbiased opinion as a student of public health. By acknowledging these factors, we aim to enhance transparency and provide readers with insights into the potential subjectivity and influence of the researchers on the research process and findings.

## Consent

Written informed consent was collected from all the participants in the study.

## Supporting information

S1 File InDepth Interview tools for RD Stakeholders. (DOCX)

S2 File Medial Analysis details files. This includes lists of all articles included in the media analysis for different stakeholders.
(DOCX)

S3 File Details of codes under each category extracted from the qualitative semi structured interviews. (DOCX)

S4 File Rare Disease Stakeholder analysis framework with scores assigned to individual stakeholders for the four characteristics Knowledge, Interest, Position and Power as described in the methods section. (XLSX)

S5 File Example Scoring Chart of Stakeholder. (XLSX)
