# Supplementary material for: Mapping the rare disease stakeholders in India
Source: PLOS Glob Public Health. 2026 Mar 26;6(3):e0003516. doi: 10.1371/journal.pgph.0003516 (PMC13020829; doi:10.1371/journal.pgph.0003516)
Supplement: S1 File — (DOCX) [file pgph.0003516.s001.docx]

**Annexure A**

**Interview guide for stakeholder interviews in Rare Disease: Policy and Public health (RDPP) Project**

***I.***         ***Stakeholder description***

1. Name

2. Designation and organization

Type of organization: Government/ Not for profit/For profit

`

***II.***         ***Experience with Rare diseases (RDs)***

3. Can you share with us how you/ your organization are associated with RDs?

4. When was the first time you heard the term RD and in what context? How did you begin working with RDs? Can you share a bit about this?

5. Have RDs in any way affected or impacted your life/work? Is it related to health-related quality of life (HRQL) or in some other way?

6. What are the key challenges you have faced in the RD ecosystem in India? As you are actively engaged in the RD ecosystem do you see any attempts being made to address some of them in our country by the government or by other bodies.

***III.***         ***Perspectives on RDs and related policies***

7. In your experience so far, do you think RD related awareness among public and government has increased in recent years? If so, what are the key events that you think may have driven this change.

8. How do you think the National Policy for Rare Diseases (NPRD) is going to address some of the challenges existing in the RD ecosystem?

1. In terms of Orphan Drugs, what do you think are the major areas of concerns that needs to be addressed. Do you think the NPRD or any other existing policies have addressed these issues in Indian context?

10.  Apart from NPRD, the National Health Policy, Disability Act Rights and New Drug and Clinical trial Rules also carried some clauses concerned with the RD patients or drugs. What are the other policies and laws that according to you have impacted or has the potential to impact the RD ecosystem in the country and how? Have any of these policies/laws impacted you or your organizations, in any way?

***IV.***         ***Influence on RD related policies***

11.  Were you/ your organization involved in the formulation or review process of the NPRD? If so in what capacity and what were your major focus and contributions in the policy?

12.  Did you/ your organization participate or get involved in any other policy which might have potential impact on the RD ecosystem of the country? If so in what capacity and what were the contributions?

13.  If ‘No’ in question 13, 14. How do you think these policies could have benefitted if there had been more inclusive channels for participation from you or similar such actors?

14.  What are the important policy directives that you think should be adopted to improve the RD ecosystem in the country?

***V.***         ***Alliance***

15. Do you think interoperability between different policies would be beneficial and how do you think it can be achieved? For example, a health policy, a RD policy, and a Drug and Clinical trial law all work in silos at present. However, if they were to have some mechanism of working together how do you think it would have benefitted the RD ecosystem of the country?

16. Do you have collaborations with other RD actors or organizations at national and/or international level? If so, what is the nature of these collaborations and how does it help to improve achieve your goals and contribute towards strengthening the larger RD ecosystem of the country.

17. Can you share a recent example of interinstitutional collaboration that has played an important role in strengthening the RD ecosystem of the country?

18. What is your assessment about international collaboration of different RD actors/ organizations in India? Do you think it is fairly common for an RD actor/organisation to have functional international collaboration? If not, what do you think are the underlying barriers?

***VI.***         ***Resources***

19. What are the resources that you or your organization can contribute towards building a stronger RD ecosystem or in strengthening the existing policy environment.

***VII.***         ***Public health perspective***

20. Tell us what are some of the resources or assets that exist in the public health system that might be able to help meet the needs and concerns of RD patients?

21. In your perspective how can we integrate public health approaches for RD management in the country? Any such best practices from other countries that you are aware of which could be implemented in India.

**Implementation**

Do you think your organization can play an important role in implementation of the policy?

8. How would you rate your organisation’s level of interest in implementing the policy?

- No or minimum interest
- Limited interest
- General interest
- High interest
- Primary interest

How do you think the implementation of NPRD (other core policy) will affect your organisation?

- It will provide opportunity
- It will cause disadvantage
- It doesn’t affect you/your organization directly.

In each case do you think the effect will be a short term or long term?

13. We talked about where your organisation stands, but having all these in mind, what is your organisation’s attitude towards NPRD and other policies we

discussed? What actions have your organisation taken to demonstrate support or

opposition for the policy?

 Extent to which they support/oppose.

- Supporter
- Moderate Supporter
- Neutral
- Moderate Opponent
- Opponent

15. What do you think are the challenges of implementing NPRD?

16. What recommendation can you provide to improve NPRD implementation?

***VIII. Any other issues that you would like to address?***
