# Supplementary material for: Mapping the rare disease stakeholders in India
Source: PLOS Glob Public Health. 2026 Mar 26;6(3):e0003516. doi: 10.1371/journal.pgph.0003516 (PMC13020829; doi:10.1371/journal.pgph.0003516)
Supplement: S2 File — This includes lists of all articles included in the media analysis for different stakeholders. (DOCX) [file pgph.0003516.s002.docx]

**2017**

1. **Keyword - MOHFW Rare disease policy India; Year - 22 March 2017 to 31 Dec 2017**

**Link :** [**https://www.google.com/search?q=MOHFW+Rare+disease+policy+india&lr=&safe=images&tbs=cdr:1,cd_min:3/22/2017,cd_max:12/31/2017&sxsrf=ALiCzsYdKbyk3L_M01TSA4EeSwfISM72PQ:1666073484694&source=lnms&tbm=nws&sa=X&ved=2ahUKEwj-8uWxj-n6AhXKEVkFHWkTCYsQ_AUoAXoECAIQAw&biw=1440&bih=789&dpr=1**](https://www.google.com/search?q=MOHFW+Rare+disease+policy+india&lr=&safe=images&tbs=cdr:1,cd_min:3/22/2017,cd_max:12/31/2017&sxsrf=ALiCzsYdKbyk3L_M01TSA4EeSwfISM72PQ:1666073484694&source=lnms&tbm=nws&sa=X&ved=2ahUKEwj-8uWxj-n6AhXKEVkFHWkTCYsQ_AUoAXoECAIQAw&biw=1440&bih=789&dpr=1)

**Remarks : 3 Relevant articles**

1. [**https://main.mohfw.gov.in/sites/default/files/Rare%20Diseases%20Policy%20FINAL.pdf**](https://main.mohfw.gov.in/sites/default/files/Rare%20Diseases%20Policy%20FINAL.pdf)
2. [**https://www.bmj.com/content/364/bmj.k5396**](https://www.bmj.com/content/364/bmj.k5396)
3. [**https://www.hindustantimes.com/health-and-fitness/facts-motive-significance-and-more-all-you-need-to-know-about-rare-disease-day/story-DqdaY06VCao1Zddj4c8FCI.html**](https://www.hindustantimes.com/health-and-fitness/facts-motive-significance-and-more-all-you-need-to-know-about-rare-disease-day/story-DqdaY06VCao1Zddj4c8FCI.html)
4. **Keyword - Niti Aayog Rare disease policy India; Year - 22 March 2017 to 31 Dec 2017**

**Link :**

[**https://www.google.com/search?q=Niti+aayog+Rare+disease+policy+india&lr=&safe=images&tbs=cdr:1,cd_min:3/22/2017,cd_max:12/31/2017&tbm=nws&sxsrf=ALiCzsa5J-KNp1FRM10xxmMJjJVILf7Meg:1666074073582&ei=2UVOY9SDI6ebseMPhumj6AE&start=20&sa=N&ved=2ahUKEwiU1MzKken6AhWnTWwGHYb0CB04ChDy0wN6BAgEEAc&biw=1440&bih=789&dpr=1**](https://www.google.com/search?q=Niti+aayog+Rare+disease+policy+india&lr=&safe=images&tbs=cdr:1,cd_min:3/22/2017,cd_max:12/31/2017&tbm=nws&sxsrf=ALiCzsa5J-KNp1FRM10xxmMJjJVILf7Meg:1666074073582&ei=2UVOY9SDI6ebseMPhumj6AE&start=20&sa=N&ved=2ahUKEwiU1MzKken6AhWnTWwGHYb0CB04ChDy0wN6BAgEEAc&biw=1440&bih=789&dpr=1)

**Remarks :**

**1.** [**https://thewire.in/health/rare-disease-policy**](https://thewire.in/health/rare-disease-policy)

**2.**[**https://timesofindia.indiatimes.com/city/bengaluru/raring-to-go-patients-to-raise-awareness-via-7000-metre-race/articleshow/57193858.cms**](https://timesofindia.indiatimes.com/city/bengaluru/raring-to-go-patients-to-raise-awareness-via-7000-metre-race/articleshow/57193858.cms)

**3.**

1. **Keyword - Ministry of finance Rare disease policy India; Year - 22 March 2017 to 31 Dec 2017**

**Link :**

[**https://www.google.com/search?q=Ministry+of+finance+Rare+disease+policy+india&lr=&safe=images&tbs=cdr:1,cd_min:3/22/2017,cd_max:12/31/2017&tbm=nws&sxsrf=ALiCzsZMP6lQlkqm2USqnVW_LPpi3D9cNw:1666074332494&ei=3EZOY_vkHYeXseMP7M6E0Aw&start=0&sa=N&ved=2ahUKEwj7wofGkun6AhWHS2wGHWwnAco4KBDy0wN6BAgBEAQ&biw=1440&bih=789&dpr=1**](https://www.google.com/search?q=Ministry+of+finance+Rare+disease+policy+india&lr=&safe=images&tbs=cdr:1,cd_min:3/22/2017,cd_max:12/31/2017&tbm=nws&sxsrf=ALiCzsZMP6lQlkqm2USqnVW_LPpi3D9cNw:1666074332494&ei=3EZOY_vkHYeXseMP7M6E0Aw&start=0&sa=N&ved=2ahUKEwj7wofGkun6AhWHS2wGHWwnAco4KBDy0wN6BAgBEAQ&biw=1440&bih=789&dpr=1)

**Remarks: 6 relevant articles found from 5 pages**

1. [**https://thewire.in/health/rare-disease-policy**](https://thewire.in/health/rare-disease-policy)
2. [**https://www.business-standard.com/article/companies/orphan-drugs-specialist-shire-to-expand-india-presence-117120701118_1.html**](https://www.business-standard.com/article/companies/orphan-drugs-specialist-shire-to-expand-india-presence-117120701118_1.html)
3. [**https://www.biospectrumindia.com/news/22/9603/karnataka-pushes-biotech-investment.html**](https://www.biospectrumindia.com/news/22/9603/karnataka-pushes-biotech-investment.html)
4. [**https://www.deccanchronicle.com/nation/current-affairs/200817/tamil-nadu-doctors-struggle-to-treat-rare-diseases.html**](https://www.deccanchronicle.com/nation/current-affairs/200817/tamil-nadu-doctors-struggle-to-treat-rare-diseases.html)
5. [**https://www.hindustantimes.com/jaipur/lsdss-demands-corpus-fund-for-diagnosis-and-treatment-of-rare-disease/story-4VIuDbVow4IUdF3C3kJW7K.html**](https://www.hindustantimes.com/jaipur/lsdss-demands-corpus-fund-for-diagnosis-and-treatment-of-rare-disease/story-4VIuDbVow4IUdF3C3kJW7K.html)
6. [**https://indianexpress.com/article/lifestyle/health/indians-prone-to-rare-genetic-diseases-study-4757874/**](https://indianexpress.com/article/lifestyle/health/indians-prone-to-rare-genetic-diseases-study-4757874/)

**4. Keyword - CDSCO Rare disease policy India; Year - 22 March 2017 to 31 Dec 2017**

**Link:** [**https://www.google.com/search?q=CDSCO+Rare+disease+policy+india&lr=&safe=images&tbs=cdr:1,cd_min:3/22/2017,cd_max:12/31/2017&tbm=nws&sxsrf=ALiCzsYpsO1Cy--2NbwM5U1yBB87UrW2XA:1666074818100&ei=wkhOY93aBaaWseMPnsea6Ak&start=0&sa=N&ved=2ahUKEwjdw86tlOn6AhUmS2wGHZ6jBp04FBDy0wN6BAgBEAQ&biw=1440&bih=789&dpr=1**](https://www.google.com/search?q=CDSCO+Rare+disease+policy+india&lr=&safe=images&tbs=cdr:1,cd_min:3/22/2017,cd_max:12/31/2017&tbm=nws&sxsrf=ALiCzsYpsO1Cy--2NbwM5U1yBB87UrW2XA:1666074818100&ei=wkhOY93aBaaWseMPnsea6Ak&start=0&sa=N&ved=2ahUKEwjdw86tlOn6AhUmS2wGHZ6jBp04FBDy0wN6BAgBEAQ&biw=1440&bih=789&dpr=1)

**Remarks : No relevant articles found**

**5. Keyword - DCGI Rare disease policy India; Year - 22 March 2017 to 31 Dec 2017**

**Link:**

[**https://www.google.com/search?q=DCGI+Rare+disease+policy+india&lr=&safe=images&tbs=cdr:1,cd_min:3/22/2017,cd_max:12/31/2017&tbm=nws&sxsrf=ALiCzsZNWgLbE6WJ7r8JBvC9149EuDzy1Q:1666075021488&ei=jUlOY620HdSKseMP3cwF&start=0&sa=N&ved=2ahUKEwjtrsyOlen6AhVURWwGHV1mAQA4HhDy0wN6BAgEEAQ&biw=1440&bih=789&dpr=1**](https://www.google.com/search?q=DCGI+Rare+disease+policy+india&lr=&safe=images&tbs=cdr:1,cd_min:3/22/2017,cd_max:12/31/2017&tbm=nws&sxsrf=ALiCzsZNWgLbE6WJ7r8JBvC9149EuDzy1Q:1666075021488&ei=jUlOY620HdSKseMP3cwF&start=0&sa=N&ved=2ahUKEwjtrsyOlen6AhVURWwGHV1mAQA4HhDy0wN6BAgEEAQ&biw=1440&bih=789&dpr=1)

**Remarks : No relevant articles found**

**6. Keyword - Court Rare disease policy India; Year - 22 March 2017 to 31 Dec 2017**

**Link:**

[**https://www.google.com/search?q=court+Rare+disease+policy+india&lr=&safe=images&tbs=cdr:1,cd_min:3/22/2017,cd_max:12/31/2017&tbm=nws&sxsrf=ALiCzsZF5PUte_o8OdK-C6M0C4TwUgjn7g:1666075665405&ei=EUxOY-anGKaWseMP0-eYuAw&start=0&sa=N&ved=2ahUKEwim9NHBl-n6AhUmS2wGHdMzBsc4KBDy0wN6BAgBEAQ&biw=1440&bih=789&dpr=1**](https://www.google.com/search?q=court+Rare+disease+policy+india&lr=&safe=images&tbs=cdr:1,cd_min:3/22/2017,cd_max:12/31/2017&tbm=nws&sxsrf=ALiCzsZF5PUte_o8OdK-C6M0C4TwUgjn7g:1666075665405&ei=EUxOY-anGKaWseMP0-eYuAw&start=0&sa=N&ved=2ahUKEwim9NHBl-n6AhUmS2wGHdMzBsc4KBDy0wN6BAgBEAQ&biw=1440&bih=789&dpr=1)

**Remarks : 2 relevant articles found, 1 duplicate from (1.) from 5 pages**

1. [**https://www.thehindu.com/sci-tech/health/when-immunity-goes-awry/article19095722.ece**](https://www.thehindu.com/sci-tech/health/when-immunity-goes-awry/article19095722.ece)
2. [**https://thewire.in/health/rare-disease-policy**](https://thewire.in/health/rare-disease-policy)

**7. Keyword - Karnataka Rare disease policy India; Year - 22 March 2017 to 31 Dec 2017**

**Link:**

[**https://www.google.com/search?q=Karnataka+Rare+disease+policy+india&lr=&safe=images&tbs=cdr:1,cd_min:3/22/2017,cd_max:12/31/2017&tbm=nws&sxsrf=ALiCzsZywuIb2yFDJg53RQ0hpdqoP13BVg:1666076115874&ei=001OY-aANfSMseMPhPGrmAI&start=0&sa=N&ved=2ahUKEwimtriYmen6AhV0RmwGHYT4CiM4KBDy0wN6BAgBEAQ&biw=1440&bih=789&dpr=1**](https://www.google.com/search?q=Karnataka+Rare+disease+policy+india&lr=&safe=images&tbs=cdr:1,cd_min:3/22/2017,cd_max:12/31/2017&tbm=nws&sxsrf=ALiCzsZywuIb2yFDJg53RQ0hpdqoP13BVg:1666076115874&ei=001OY-aANfSMseMPhPGrmAI&start=0&sa=N&ved=2ahUKEwimtriYmen6AhV0RmwGHYT4CiM4KBDy0wN6BAgBEAQ&biw=1440&bih=789&dpr=1)

**Remarks: 2 relevant articles found from 5 pages**

1. [**https://www.biospectrumindia.com/news/23/9223/gsk-pharma-plans-investment-in-karnataka-plant.html**](https://www.biospectrumindia.com/news/23/9223/gsk-pharma-plans-investment-in-karnataka-plant.html) **- EXCLUDED**
2. [**https://www.biospectrumindia.com/news/22/9301/karnataka-govt-announces-two-flagship-events.html**](https://www.biospectrumindia.com/news/22/9301/karnataka-govt-announces-two-flagship-events.html)
3. [**https://www.thehindu.com/news/national/karnataka/new-biotech-policy-for-2017-22-gets-nod/article19764466.ece**](https://www.thehindu.com/news/national/karnataka/new-biotech-policy-for-2017-22-gets-nod/article19764466.ece) **- EXCLUDED (Duplicate - Quotes already existing)**

**8. Keyword - Kerala Rare disease policy India; Year - 22 March 2017 to 31 Dec 2017**

**Link:**

[**https://www.google.com/search?q=Kerala+Rare+disease+policy+india&lr=&safe=images&tbs=cdr:1,cd_min:3/22/2017,cd_max:12/31/2017&tbm=nws&sxsrf=ALiCzsa3f5P1wKMYofwZxqbKwBqRKEazoA:1666076325375&ei=pU5OY87CFvuMseMPz-KbyA4&start=0&sa=N&ved=2ahUKEwiOqav8men6AhV7RmwGHU_xBuk4KBDy0wN6BAgCEAQ&biw=1440&bih=789&dpr=1**](https://www.google.com/search?q=Kerala+Rare+disease+policy+india&lr=&safe=images&tbs=cdr:1,cd_min:3/22/2017,cd_max:12/31/2017&tbm=nws&sxsrf=ALiCzsa3f5P1wKMYofwZxqbKwBqRKEazoA:1666076325375&ei=pU5OY87CFvuMseMPz-KbyA4&start=0&sa=N&ved=2ahUKEwiOqav8men6AhV7RmwGHU_xBuk4KBDy0wN6BAgCEAQ&biw=1440&bih=789&dpr=1)

**Remarks: 3 relevant articles found from 5 pages**

1. [**https://www.newindianexpress.com/states/kerala/2017/oct/27/underprivileged-kerala-boy-suffering-from-rare-disease-is-on-a-mission-find-cures-through-genetic-re-1684531.html**](https://www.newindianexpress.com/states/kerala/2017/oct/27/underprivileged-kerala-boy-suffering-from-rare-disease-is-on-a-mission-find-cures-through-genetic-re-1684531.html) [**https://www.thehindu.com/opinion/op-ed/the-interpretation-of-a-malady/article18027504.ece**](https://www.thehindu.com/opinion/op-ed/the-interpretation-of-a-malady/article18027504.ece) **- EXCLUDED (Story of a single pt - no quotes from listed stakeholders)**
2. [**https://www.vccircle.com/medgenome-raises-30-mn-from-sequoia-sofina-in-series-c-funding**](https://www.vccircle.com/medgenome-raises-30-mn-from-sequoia-sofina-in-series-c-funding)

**CODING DONE**

**2018**

1. **Keyword - MOHFW Rare disease policy India; Year - 1 Jan 2018 to 31 Dec 2018**

**Link :** [**https://www.google.com/search?q=MOHFW+Rare+disease+policy+india&lr=&safe=images&biw=1440&bih=789&sxsrf=ALiCzsYQI_zvjSvDLDDPqEN1wXevGr3IIg%3A1666854070548&source=lnt&tbs=cdr%3A1%2Ccd_min%3A1%2F1%2F2018%2Ccd_max%3A12%2F31%2F2018&tbm=nws**](https://www.google.com/search?q=MOHFW+Rare+disease+policy+india&lr=&safe=images&biw=1440&bih=789&sxsrf=ALiCzsYQI_zvjSvDLDDPqEN1wXevGr3IIg%3A1666854070548&source=lnt&tbs=cdr%3A1%2Ccd_min%3A1%2F1%2F2018%2Ccd_max%3A12%2F31%2F2018&tbm=nws)

**Remarks : No relevant articles found**

1. **Keyword - Niti Aayog Rare disease policy India; Year -1 Jan 2018 to 31 Dec 2018**

**Link :**

[**https://www.google.com/search?q=Niti+aayog+Rare+disease+policy+india&lr=&safe=images&biw=1440&bih=789&sxsrf=ALiCzsbuif0HzXsxFuHVjxhe9pBEbpPtLQ%3A1666854182629&source=lnt&tbs=cdr%3A1%2Ccd_min%3A1%2F1%2F2018%2Ccd_max%3A12%2F31%2F2018&tbm=nws**](https://www.google.com/search?q=Niti+aayog+Rare+disease+policy+india&lr=&safe=images&biw=1440&bih=789&sxsrf=ALiCzsbuif0HzXsxFuHVjxhe9pBEbpPtLQ%3A1666854182629&source=lnt&tbs=cdr%3A1%2Ccd_min%3A1%2F1%2F2018%2Ccd_max%3A12%2F31%2F2018&tbm=nws)

**Remarks : No relevant articles found**

1. **Keyword - Ministry of finance Rare disease policy India; Year - 1 Jan 2018 to 31 Dec 2018**

**Link :**

[**https://www.google.com/search?q=Ministry+of+finance+Rare+disease+policy+india&lr=&safe=images&tbs=cdr:1,cd_min:3/22/2017,cd_max:12/31/2017&tbm=nws&sxsrf=ALiCzsZMP6lQlkqm2USqnVW_LPpi3D9cNw:1666074332494&ei=3EZOY_vkHYeXseMP7M6E0Aw&start=0&sa=N&ved=2ahUKEwj7wofGkun6AhWHS2wGHWwnAco4KBDy0wN6BAgBEAQ&biw=1440&bih=789&dpr=1**](https://www.google.com/search?q=Ministry+of+finance+Rare+disease+policy+india&lr=&safe=images&tbs=cdr:1,cd_min:3/22/2017,cd_max:12/31/2017&tbm=nws&sxsrf=ALiCzsZMP6lQlkqm2USqnVW_LPpi3D9cNw:1666074332494&ei=3EZOY_vkHYeXseMP7M6E0Aw&start=0&sa=N&ved=2ahUKEwj7wofGkun6AhWHS2wGHWwnAco4KBDy0wN6BAgBEAQ&biw=1440&bih=789&dpr=1)

**Remarks: 3 relevant articles found from 5 pages**

1. [**https://scroll.in/pulse/906885/longer-wait-for-patients-as-the-government-calls-its-own-rare-disease-policy-impractical**](https://scroll.in/pulse/906885/longer-wait-for-patients-as-the-government-calls-its-own-rare-disease-policy-impractical)
2. [**https://www.biospectrumindia.com/features/17/10498/world-rare-disease-day-prevention-of-sickle-cell-disease-early-screening-and-awareness-a-must.html**](https://www.biospectrumindia.com/features/17/10498/world-rare-disease-day-prevention-of-sickle-cell-disease-early-screening-and-awareness-a-must.html) **- EXCLUDED (Sicke cell anemia)**
3. [**https://www.biospectrumindia.com/news/22/10514/delhi-commemorates-rare-disease-day.html**](https://www.biospectrumindia.com/news/22/10514/delhi-commemorates-rare-disease-day.html)

**4. Keyword - CDSCO Rare disease policy India; Year -1 Jan 2018 to 31 Dec 2018**

**Link:** [**https://www.google.com/search?q=CDSCO+Rare+disease+policy+india&lr=&safe=images&biw=1440&bih=789&sxsrf=ALiCzsZ6Aj5zZclcc9-ecvR-OSiJXwL2TQ%3A1667024227666&source=lnt&tbs=cdr%3A1%2Ccd_min%3A1%2F1%2F2018%2Ccd_max%3A12%2F31%2F2018&tbm=nws**](https://www.google.com/search?q=CDSCO+Rare+disease+policy+india&lr=&safe=images&biw=1440&bih=789&sxsrf=ALiCzsZ6Aj5zZclcc9-ecvR-OSiJXwL2TQ%3A1667024227666&source=lnt&tbs=cdr%3A1%2Ccd_min%3A1%2F1%2F2018%2Ccd_max%3A12%2F31%2F2018&tbm=nws)

**Remarks : 2 relevant articles found**

1. [**https://www.clinicalleader.com/doc/india-making-way-for-separate-clinical-trials-rules-0001**](https://www.clinicalleader.com/doc/india-making-way-for-separate-clinical-trials-rules-0001) **- No quotes , more like a review of new CT rules**
2. [**https://www.thehindu.com/sci-tech/health/rare-and-ignored/article22538355.ece**](https://www.thehindu.com/sci-tech/health/rare-and-ignored/article22538355.ece) **- No quotes**

**5. Keyword - DCGI Rare disease policy India; Year - 1 Jan 2018 to 31 Dec 2018**

**Link:**

[**https://www.google.com/search?q=DCGI+Rare+disease+policy+india&lr=&safe=images&biw=1440&bih=789&sxsrf=ALiCzsarSXiDJ790OYmQEFyQ_lzAYp2CYg%3A1667024171023&source=lnt&tbs=cdr%3A1%2Ccd_min%3A1%2F1%2F2018%2Ccd_max%3A12%2F31%2F2018&tbm=nws**](https://www.google.com/search?q=DCGI+Rare+disease+policy+india&lr=&safe=images&biw=1440&bih=789&sxsrf=ALiCzsarSXiDJ790OYmQEFyQ_lzAYp2CYg%3A1667024171023&source=lnt&tbs=cdr%3A1%2Ccd_min%3A1%2F1%2F2018%2Ccd_max%3A12%2F31%2F2018&tbm=nws)

**Remarks : No relevant articles found**

**6. Keyword - Court Rare disease policy India; Year - 1 Jan 2018 to 31 Dec 2018**

**Link:**

[**https://www.google.com/search?q=court+Rare+disease+policy+india&lr=&safe=images&biw=1440&bih=789&sxsrf=ALiCzsbTY8HVeXg7fVA1K-4rSfhtnDCS4g%3A1667024332855&source=lnt&tbs=cdr%3A1%2Ccd_min%3A1%2F1%2F2018%2Ccd_max%3A12%2F31%2F2018&tbm=nws**](https://www.google.com/search?q=court+Rare+disease+policy+india&lr=&safe=images&biw=1440&bih=789&sxsrf=ALiCzsbTY8HVeXg7fVA1K-4rSfhtnDCS4g%3A1667024332855&source=lnt&tbs=cdr%3A1%2Ccd_min%3A1%2F1%2F2018%2Ccd_max%3A12%2F31%2F2018&tbm=nws)

**Remarks : 4 relevant articles found**

1. [**https://www.downtoearth.org.in/news/health/india-s-rare-diseases-policy-hangs-in-balance-62611**](https://www.downtoearth.org.in/news/health/india-s-rare-diseases-policy-hangs-in-balance-62611)
2. [**https://thewire.in/health/coverage-genetic-diseases-welcomed-activities-await-new-insurance-framework**](https://thewire.in/health/coverage-genetic-diseases-welcomed-activities-await-new-insurance-framework)
3. [**https://www.newindianexpress.com/states/karnataka/2018/mar/22/insurers-cannot-reject-health-claims-based-on-genetic-disorders-says-irdai-1790724.html**](https://www.newindianexpress.com/states/karnataka/2018/mar/22/insurers-cannot-reject-health-claims-based-on-genetic-disorders-says-irdai-1790724.html) **- np relevant quotes**
4. [**https://bangaloremirror.indiatimes.com/opinion/sunday-read/bengaluru-ngo-files-a-pil-in-the-supreme-court-to-help-patients-suffering-from-rare-diseases/articleshow/66298383.cms**](https://bangaloremirror.indiatimes.com/opinion/sunday-read/bengaluru-ngo-files-a-pil-in-the-supreme-court-to-help-patients-suffering-from-rare-diseases/articleshow/66298383.cms) **- All quotes by Prasanna Shirol**

**7. Keyword - Karnataka Rare disease policy India; Year - 1 Jan 2018 to 31 Dec 2018**

**Link:**

[**https://www.google.com/search?q=Karnataka+Rare+disease+policy+india&lr=&safe=images&biw=1440&bih=789&sxsrf=ALiCzsYtkcC-0eelUjogknDQxj7NZJjHRg%3A1667026636507&source=lnt&tbs=cdr%3A1%2Ccd_min%3A1%2F1%2F2018%2Ccd_max%3A12%2F31%2F2018&tbm=nws**](https://www.google.com/search?q=Karnataka+Rare+disease+policy+india&lr=&safe=images&biw=1440&bih=789&sxsrf=ALiCzsYtkcC-0eelUjogknDQxj7NZJjHRg%3A1667026636507&source=lnt&tbs=cdr%3A1%2Ccd_min%3A1%2F1%2F2018%2Ccd_max%3A12%2F31%2F2018&tbm=nws)

**Remarks: 1 relevant article found from 5 pages**

1. [**https://bangaloremirror.indiatimes.com/opinion/sunday-read/these-two-collegemates-are-making-drugs-for-rare-diseases-that-afflict-mostly-children/articleshow/64809685.cms**](https://bangaloremirror.indiatimes.com/opinion/sunday-read/these-two-collegemates-are-making-drugs-for-rare-diseases-that-afflict-mostly-children/articleshow/64809685.cms) **- not able to copy paste**

**8. Keyword - Kerala Rare disease policy India; Year -1 Jan 2018 to 31 Dec 2018**

**Link:**

[**https://www.google.com/search?q=Kerala+Rare+disease+policy+india&lr=&safe=images&biw=1440&bih=789&sxsrf=ALiCzsYU1STD4aUF29ENZTO0VzeSVkzWxg%3A1667026605577&source=lnt&tbs=cdr%3A1%2Ccd_min%3A1%2F1%2F2018%2Ccd_max%3A12%2F31%2F2018&tbm=nws**](https://www.google.com/search?q=Kerala+Rare+disease+policy+india&lr=&safe=images&biw=1440&bih=789&sxsrf=ALiCzsYU1STD4aUF29ENZTO0VzeSVkzWxg%3A1667026605577&source=lnt&tbs=cdr%3A1%2Ccd_min%3A1%2F1%2F2018%2Ccd_max%3A12%2F31%2F2018&tbm=nws)

**Remarks: `1 relevant article found from 5 pages**

1. [**https://www.telegraphindia.com/health/govt-does-a-u-turn-on-medical-fund-for-kids-with-rare-diseases-that-are-expensive-to-treat/cid/1679330**](https://www.telegraphindia.com/health/govt-does-a-u-turn-on-medical-fund-for-kids-with-rare-diseases-that-are-expensive-to-treat/cid/1679330) **- Quotes repeating from previous article**

**CODING DONE**

**2019**

1. **Keyword - MOHFW Rare disease policy India; Year - 1 Jan 2019 to 31 Dec 2019**

**Link :** [**https://www.google.com/search?q=MOHFW+Rare+disease+policy+india&lr=&safe=images&biw=1440&bih=789&sxsrf=ALiCzsbUIXWBUZiXdcVp0VRrY7XwC7jnXg%3A1667025105252&source=lnt&tbs=cdr%3A1%2Ccd_min%3A1%2F1%2F2019%2Ccd_max%3A12%2F31%2F2019&tbm=nws**](https://www.google.com/search?q=MOHFW+Rare+disease+policy+india&lr=&safe=images&biw=1440&bih=789&sxsrf=ALiCzsbUIXWBUZiXdcVp0VRrY7XwC7jnXg%3A1667025105252&source=lnt&tbs=cdr%3A1%2Ccd_min%3A1%2F1%2F2019%2Ccd_max%3A12%2F31%2F2019&tbm=nws)

1. [**https://www.raps.org/news-and-articles/news-articles/2019/7/indias-new-drugs-and-clinical-trials-rules-an-in**](https://www.raps.org/news-and-articles/news-articles/2019/7/indias-new-drugs-and-clinical-trials-rules-an-in) **- No quotes**
2. [**https://www.mondaq.com/india/life-sciences-biotechnology-nanotechnology/779544/patented-new-drugs-and-orphan-drugs-out-of-price-control-in-india**](https://www.mondaq.com/india/life-sciences-biotechnology-nanotechnology/779544/patented-new-drugs-and-orphan-drugs-out-of-price-control-in-india) **- No quotes**

**Remarks : 2 relevant articles found**

1. **Keyword - Niti Aayog Rare disease policy India; Year -1 Jan 2021 to 31 Dec 2021**

**Link :**

[**https://www.google.com/search?q=Niti+aayog+Rare+disease+policy+india&lr=&safe=images&biw=1440&bih=789&sxsrf=ALiCzsbXWK-v4rvtkrh3MEGRGAQO1lTaUw%3A1667025284206&source=lnt&tbs=cdr%3A1%2Ccd_min%3A1%2F1%2F2019%2Ccd_max%3A12%2F31%2F2019&tbm=nws**](https://www.google.com/search?q=Niti+aayog+Rare+disease+policy+india&lr=&safe=images&biw=1440&bih=789&sxsrf=ALiCzsbXWK-v4rvtkrh3MEGRGAQO1lTaUw%3A1667025284206&source=lnt&tbs=cdr%3A1%2Ccd_min%3A1%2F1%2F2019%2Ccd_max%3A12%2F31%2F2019&tbm=nws)

**Remarks : No relevant articles found**

1. **Keyword - Ministry of finance Rare disease policy India; Year - 1 Jan 2019 to 31 Dec 2019**

**Link :**

[**https://www.google.com/search?q=Ministry+of+finance+Rare+disease+policy+india&lr=&safe=images&biw=1440&bih=789&sxsrf=ALiCzsYweBkY1t3S8g7Zh3M08RJp6aK1aw%3A1667026287066&source=lnt&tbs=cdr%3A1%2Ccd_min%3A1%2F1%2F2019%2Ccd_max%3A12%2F31%2F2019&tbm=nws**](https://www.google.com/search?q=Ministry+of+finance+Rare+disease+policy+india&lr=&safe=images&biw=1440&bih=789&sxsrf=ALiCzsYweBkY1t3S8g7Zh3M08RJp6aK1aw%3A1667026287066&source=lnt&tbs=cdr%3A1%2Ccd_min%3A1%2F1%2F2019%2Ccd_max%3A12%2F31%2F2019&tbm=nws)

**Remarks: 3 relevant articles found from 5 pages**

1. [**https://www.hindustantimes.com/india-news/health-ministry-plans-policy-for-rare-diseases-treatment/story-2yzhRglwvw8qU0bazHokqN.html**](https://www.hindustantimes.com/india-news/health-ministry-plans-policy-for-rare-diseases-treatment/story-2yzhRglwvw8qU0bazHokqN.html)
2. [**https://www.biospectrumindia.com/news/22/14012/health-minister-expedites-process-of-rare-disease-policy-formation.html**](https://www.biospectrumindia.com/news/22/14012/health-minister-expedites-process-of-rare-disease-policy-formation.html)
3. [**https://indianexpress.com/article/lifestyle/health/enzyme-replacement-therapy-may-extend-lifespan-of-rare-disease-patients-5832357/**](https://indianexpress.com/article/lifestyle/health/enzyme-replacement-therapy-may-extend-lifespan-of-rare-disease-patients-5832357/) **- No relevant quotes**

**4. Keyword - CDSCO Rare disease policy India; Year - 1 Jan 2019 to 31 Dec 2019**

**Link:** [**https://www.google.com/search?q=CDSCO+Rare+disease+policy+india&lr=&safe=images&biw=1440&bih=789&sxsrf=ALiCzsbTuuw-Z5repqwypB-jB3Jj4TYnsw%3A1667026176607&source=lnt&tbs=cdr%3A1%2Ccd_min%3A1%2F1%2F2019%2Ccd_max%3A12%2F31%2F2019&tbm=nws**](https://www.google.com/search?q=CDSCO+Rare+disease+policy+india&lr=&safe=images&biw=1440&bih=789&sxsrf=ALiCzsbTuuw-Z5repqwypB-jB3Jj4TYnsw%3A1667026176607&source=lnt&tbs=cdr%3A1%2Ccd_min%3A1%2F1%2F2019%2Ccd_max%3A12%2F31%2F2019&tbm=nws)

**Remarks : 3 relevant articles found**

1. [**https://theprint.in/health/genetics-labs-come-under-watch-govt-to-monitor-gene-therapy-research-across-india/276840/**](https://theprint.in/health/genetics-labs-come-under-watch-govt-to-monitor-gene-therapy-research-across-india/276840/)
2. [**https://www.downtoearth.org.in/blog/health/encouraging-orphan-drugs-in-india-67581**](https://www.downtoearth.org.in/blog/health/encouraging-orphan-drugs-in-india-67581) **- No quotes**
3. [**https://www.hindustantimes.com/india-news/gene-editing-medical-board-drafts-new-laws/story-WXR5RjSoMWPDcnhu9yZOtK.html**](https://www.hindustantimes.com/india-news/gene-editing-medical-board-drafts-new-laws/story-WXR5RjSoMWPDcnhu9yZOtK.html)

**5. Keyword - DCGI Rare disease policy India; Year -1 Jan 2021 to 31 Dec 2021**

**Link:**

[**https://www.google.com/search?q=DCGI+Rare+disease+policy+india&lr=&safe=images&biw=1440&bih=789&sxsrf=ALiCzsYwhmqnHxfsqfCJro0ip86n3WESUA%3A1667026332879&source=lnt&tbs=cdr%3A1%2Ccd_min%3A1%2F1%2F2019%2Ccd_max%3A12%2F31%2F2019&tbm=nws**](https://www.google.com/search?q=DCGI+Rare+disease+policy+india&lr=&safe=images&biw=1440&bih=789&sxsrf=ALiCzsYwhmqnHxfsqfCJro0ip86n3WESUA%3A1667026332879&source=lnt&tbs=cdr%3A1%2Ccd_min%3A1%2F1%2F2019%2Ccd_max%3A12%2F31%2F2019&tbm=nws)

1. [**https://www.nagpurtoday.in/incurable-dmd-disease-takes-heavy-toll-of-children-in-india/09041843**](https://www.nagpurtoday.in/incurable-dmd-disease-takes-heavy-toll-of-children-in-india/09041843) **- no quotes**

**Remarks : No relevant articles found**

**6. Keyword - Court Rare disease policy India; Year - 1 Jan 2019 to 31 Dec 2019**

**Link:**

[**https://www.google.com/search?q=court+Rare+disease+policy+india&lr=&safe=images&biw=1440&bih=789&sxsrf=ALiCzsZJ4IJbAAe0M5OyRysW6D1cF6ExCA%3A1667026352349&source=lnt&tbs=cdr%3A1%2Ccd_min%3A1%2F1%2F2019%2Ccd_max%3A12%2F31%2F2019&tbm=nws**](https://www.google.com/search?q=court+Rare+disease+policy+india&lr=&safe=images&biw=1440&bih=789&sxsrf=ALiCzsZJ4IJbAAe0M5OyRysW6D1cF6ExCA%3A1667026352349&source=lnt&tbs=cdr%3A1%2Ccd_min%3A1%2F1%2F2019%2Ccd_max%3A12%2F31%2F2019&tbm=nws)

**Remarks : 4 relevant articles found**

1. [**https://battendiseasenews.com/news/nonprofit-works-to-raise-rare-disease-awareness-in-india-connect-with-us-groups/**](https://battendiseasenews.com/news/nonprofit-works-to-raise-rare-disease-awareness-in-india-connect-with-us-groups/) **- quotes by PAGs**
2. [**https://www.thehindubusinessline.com/companies/takeda-brings-drugs-for-3-rare-diseases-to-india-but-pricing-concerns-remain/article28447477.ece**](https://www.thehindubusinessline.com/companies/takeda-brings-drugs-for-3-rare-diseases-to-india-but-pricing-concerns-remain/article28447477.ece) **- No relevant quotes**
3. [**https://www.deccanherald.com/city/govt-hospital-runs-out-of-medicines-for-rare-diseases-723948.html**](https://www.deccanherald.com/city/govt-hospital-runs-out-of-medicines-for-rare-diseases-723948.html)
4. [**https://indianexpress.com/article/cities/delhi/delhi-three-year-old-with-rare-disorder-poses-dilemma-for-hospital-5561955/**](https://indianexpress.com/article/cities/delhi/delhi-three-year-old-with-rare-disorder-poses-dilemma-for-hospital-5561955/) **- need subscription to read**

**7. Keyword - Karnataka Rare disease policy India; Year - 1 Jan 2019 to 31 Dec 2019**

**Link:**

[**https://www.google.com/search?q=Karnataka+Rare+disease+policy+india&lr=&safe=images&biw=1440&bih=789&sxsrf=ALiCzsZuYLjuKWFEg0sbdF_W-IFhGo5-uA%3A1667026381533&source=lnt&tbs=cdr%3A1%2Ccd_min%3A1%2F1%2F2019%2Ccd_max%3A12%2F31%2F2019&tbm=nws**](https://www.google.com/search?q=Karnataka+Rare+disease+policy+india&lr=&safe=images&biw=1440&bih=789&sxsrf=ALiCzsZuYLjuKWFEg0sbdF_W-IFhGo5-uA%3A1667026381533&source=lnt&tbs=cdr%3A1%2Ccd_min%3A1%2F1%2F2019%2Ccd_max%3A12%2F31%2F2019&tbm=nws)

**Remarks: 1 relevant article found from 5 pages**

1. [**https://www.deccanherald.com/national/scientists-to-take-a-shot-at-a-rare-disease-771133.html**](https://www.deccanherald.com/national/scientists-to-take-a-shot-at-a-rare-disease-771133.html) **- No relevant quotes**

**8. Keyword - Kerala Rare disease policy India; Year -1 Jan 2021 to 31 Dec 2021**

**Link:**

[**https://www.google.com/search?q=Kerala+Rare+disease+policy+india&lr=&safe=images&tbs=cdr:1,cd_min:3/22/2017,cd_max:12/31/2017&tbm=nws&sxsrf=ALiCzsa3f5P1wKMYofwZxqbKwBqRKEazoA:1666076325375&ei=pU5OY87CFvuMseMPz-KbyA4&start=0&sa=N&ved=2ahUKEwiOqav8men6AhV7RmwGHU_xBuk4KBDy0wN6BAgCEAQ&biw=1440&bih=789&dpr=1**](https://www.google.com/search?q=Kerala+Rare+disease+policy+india&lr=&safe=images&tbs=cdr:1,cd_min:3/22/2017,cd_max:12/31/2017&tbm=nws&sxsrf=ALiCzsa3f5P1wKMYofwZxqbKwBqRKEazoA:1666076325375&ei=pU5OY87CFvuMseMPz-KbyA4&start=0&sa=N&ved=2ahUKEwiOqav8men6AhV7RmwGHU_xBuk4KBDy0wN6BAgCEAQ&biw=1440&bih=789&dpr=1)

**Remarks: `1 relevant article found from 5 pages**

1. [**https://www.aninews.in/news/business/dr-lal-pathlabs-ltd-collaborates-with-ordi-to-sensitize-india-on-rare-diseases20190305161730/**](https://www.aninews.in/news/business/dr-lal-pathlabs-ltd-collaborates-with-ordi-to-sensitize-india-on-rare-diseases20190305161730/) **- No relevant quotes**

**CODING DONE**

**2020**

1. **Keyword - MOHFW Rare disease policy India; Year - 1 Jan 2019 to 31 Dec 2019**

**Link :** [**https://www.google.com/search?q=MOHFW+Rare+disease+policy+india&lr=&safe=images&biw=1440&bih=789&sxsrf=ALiCzsa4rsOytXuNM9PxQBYaI23o3MGI4Q%3A1667723055962&source=lnt&tbs=cdr%3A1%2Ccd_min%3A1%2F1%2F2020%2Ccd_max%3A12%2F31%2F2020&tbm=nws**](https://www.google.com/search?q=MOHFW+Rare+disease+policy+india&lr=&safe=images&biw=1440&bih=789&sxsrf=ALiCzsa4rsOytXuNM9PxQBYaI23o3MGI4Q%3A1667723055962&source=lnt&tbs=cdr%3A1%2Ccd_min%3A1%2F1%2F2020%2Ccd_max%3A12%2F31%2F2020&tbm=nws)

1. [**https://medicaldialogues.in/news/health/government-policies/mohfw-releases-national-policy-for-rare-diseases-check-out-salient-features-61970**](https://medicaldialogues.in/news/health/government-policies/mohfw-releases-national-policy-for-rare-diseases-check-out-salient-features-61970) **- need subscription**
2. [**https://ehealth.eletsonline.com/2020/12/vishal-chauhan-appointed-as-joint-secretary-mohfw-goi/**](https://ehealth.eletsonline.com/2020/12/vishal-chauhan-appointed-as-joint-secretary-mohfw-goi/) **- No quotes**

**Remarks : 2 relevant articles found**

1. **Keyword - Niti Aayog Rare disease policy India; Year -1 Jan 2021 to 31 Dec 2021**

**Link :**

[**https://www.google.com/search?q=Niti+aayog+Rare+disease+policy+india&lr=&safe=images&biw=1440&bih=789&sxsrf=ALiCzsY832VGoKNBjLU9MT4GO79EgiCILQ%3A1667723081495&source=lnt&tbs=cdr%3A1%2Ccd_min%3A1%2F1%2F2020%2Ccd_max%3A12%2F31%2F2020&tbm=nws**](https://www.google.com/search?q=Niti+aayog+Rare+disease+policy+india&lr=&safe=images&biw=1440&bih=789&sxsrf=ALiCzsY832VGoKNBjLU9MT4GO79EgiCILQ%3A1667723081495&source=lnt&tbs=cdr%3A1%2Ccd_min%3A1%2F1%2F2020%2Ccd_max%3A12%2F31%2F2020&tbm=nws)

**Remarks : No relevant articles found**

1. **Keyword - Ministry of finance Rare disease policy India; Year - 1 Jan 2019 to 31 Dec 2019**

**Link :**

[**https://www.google.com/search?q=Niti+aayog+Rare+disease+policy+india&lr=&safe=images&biw=1440&bih=789&sxsrf=ALiCzsY832VGoKNBjLU9MT4GO79EgiCILQ%3A1667723081495&source=lnt&tbs=cdr%3A1%2Ccd_min%3A1%2F1%2F2020%2Ccd_max%3A12%2F31%2F2020&tbm=nws**](https://www.google.com/search?q=Niti+aayog+Rare+disease+policy+india&lr=&safe=images&biw=1440&bih=789&sxsrf=ALiCzsY832VGoKNBjLU9MT4GO79EgiCILQ%3A1667723081495&source=lnt&tbs=cdr%3A1%2Ccd_min%3A1%2F1%2F2020%2Ccd_max%3A12%2F31%2F2020&tbm=nws)

1. [**https://indianexpress.com/article/explained/rare-diseases-indian-council-of-medical-research-6216254/**](https://indianexpress.com/article/explained/rare-diseases-indian-council-of-medical-research-6216254/) **- need subscription**
2. [**https://www.livemint.com/news/india/govt-may-treat-rare-disease-patients-at-one-time-treatment-cost-up-to-rs-15-lakh-11578941088572.html**](https://www.livemint.com/news/india/govt-may-treat-rare-disease-patients-at-one-time-treatment-cost-up-to-rs-15-lakh-11578941088572.html) **- no relevant quotes**
3. [**https://www.livemint.com/news/india/govt-may-treat-rare-disease-patients-at-one-time-treatment-cost-up-to-rs-15-lakh-11578941088572.html**](https://www.livemint.com/news/india/govt-may-treat-rare-disease-patients-at-one-time-treatment-cost-up-to-rs-15-lakh-11578941088572.html) **- No quotes**
4. [**https://timesofindia.indiatimes.com/blogs/voices/world-rare-disease-day/**](https://timesofindia.indiatimes.com/blogs/voices/world-rare-disease-day/) **- no quotes**

**Remarks: 4 relevant articles found from 5 pages**

**4. Keyword - CDSCO Rare disease policy India; Year - 1 Jan 2019 to 31 Dec 2019**

**Link:** [**https://www.google.com/search?q=CDSCO+Rare+disease+policy+india&lr=&safe=images&biw=1440&bih=789&sxsrf=ALiCzsaXymuV4r7TCDxbctPkl7tGfrjVNw%3A1667723369669&source=lnt&tbs=cdr%3A1%2Ccd_min%3A1%2F1%2F2020%2Ccd_max%3A12%2F31%2F2020&tbm=nws**](https://www.google.com/search?q=CDSCO+Rare+disease+policy+india&lr=&safe=images&biw=1440&bih=789&sxsrf=ALiCzsaXymuV4r7TCDxbctPkl7tGfrjVNw%3A1667723369669&source=lnt&tbs=cdr%3A1%2Ccd_min%3A1%2F1%2F2020%2Ccd_max%3A12%2F31%2F2020&tbm=nws)

**Remarks : No relevant articles found**

**5. Keyword - DCGI Rare disease policy India; Year -1 Jan 2021 to 31 Dec 2021**

**Link:**

[**https://www.google.com/search?q=DCGI+Rare+disease+policy+india&lr=&safe=images&biw=1440&bih=789&sxsrf=ALiCzsYwhmqnHxfsqfCJro0ip86n3WESUA%3A1667026332879&source=lnt&tbs=cdr%3A1%2Ccd_min%3A1%2F1%2F2019%2Ccd_max%3A12%2F31%2F2019&tbm=nws**](https://www.google.com/search?q=DCGI+Rare+disease+policy+india&lr=&safe=images&biw=1440&bih=789&sxsrf=ALiCzsYwhmqnHxfsqfCJro0ip86n3WESUA%3A1667026332879&source=lnt&tbs=cdr%3A1%2Ccd_min%3A1%2F1%2F2019%2Ccd_max%3A12%2F31%2F2019&tbm=nws)

1. [**https://www.raps.org/news-and-articles/news-articles/2019/7/indias-new-drugs-and-clinical-trials-rules-an-in**](https://www.raps.org/news-and-articles/news-articles/2019/7/indias-new-drugs-and-clinical-trials-rules-an-in) **- No quotes**
2. [**https://www.mondaq.com/india/life-sciences-biotechnology-nanotechnology/779544/patented-new-drugs-and-orphan-drugs-out-of-price-control-in-india**](https://www.mondaq.com/india/life-sciences-biotechnology-nanotechnology/779544/patented-new-drugs-and-orphan-drugs-out-of-price-control-in-india) **- No quotes**
3. [**https://www.downtoearth.org.in/blog/health/encouraging-orphan-drugs-in-india-67581**](https://www.downtoearth.org.in/blog/health/encouraging-orphan-drugs-in-india-67581) **- No quotes**

**Remarks : No relevant articles found**

**6. Keyword - Court Rare disease policy India; Year - 1 Jan 2019 to 31 Dec 2019**

**Link:**

[**https://www.google.com/search?q=Kerala+Rare+disease+policy+india&lr=&safe=images&biw=1440&bih=789&sxsrf=ALiCzsYRzSv3vrykYkCC2GPLqlbmZ86ZRA%3A1667741943308&source=lnt&tbs=cdr%3A1%2Ccd_min%3A1%2F1%2F2020%2Ccd_max%3A12%2F31%2F2020&tbm=nws**](https://www.google.com/search?q=Kerala+Rare+disease+policy+india&lr=&safe=images&biw=1440&bih=789&sxsrf=ALiCzsYRzSv3vrykYkCC2GPLqlbmZ86ZRA%3A1667741943308&source=lnt&tbs=cdr%3A1%2Ccd_min%3A1%2F1%2F2020%2Ccd_max%3A12%2F31%2F2020&tbm=nws)

**Remarks : 1 relevant article found**

1. [**https://www.thehindu.com/news/cities/Delhi/hc-asks-aiims-to-provide-free-treatment-to-18-month-old-girl-for-rare-disease/article61958440.ece**](https://www.thehindu.com/news/cities/Delhi/hc-asks-aiims-to-provide-free-treatment-to-18-month-old-girl-for-rare-disease/article61958440.ece)

**7. Keyword - Karnataka Rare disease policy India; Year - 1 Jan 2019 to 31 Dec 2019**

**Link:**

[**https://www.google.com/search?q=Karnataka+Rare+disease+policy+india&lr=&safe=images&biw=1440&bih=789&sxsrf=ALiCzsZ5h8qGcKLPyGPO91m5vJBlePFQWw%3A1667723961723&source=lnt&tbs=cdr%3A1%2Ccd_min%3A1%2F1%2F2020%2Ccd_max%3A12%2F31%2F2020&tbm=nws**](https://www.google.com/search?q=Karnataka+Rare+disease+policy+india&lr=&safe=images&biw=1440&bih=789&sxsrf=ALiCzsZ5h8qGcKLPyGPO91m5vJBlePFQWw%3A1667723961723&source=lnt&tbs=cdr%3A1%2Ccd_min%3A1%2F1%2F2020%2Ccd_max%3A12%2F31%2F2020&tbm=nws)

**Remarks: No relevant article found**

**8. Keyword - Kerala Rare disease policy India; Year -1 Jan 2019 to 31 Dec 2019**

**Link:**

[**https://www.google.com/search?q=Kerala+Rare+disease+policy+india&lr=&safe=images&biw=1440&bih=789&sxsrf=ALiCzsZGq96U-NugrG95kBtzLr6Q-0ovhg%3A1667724042701&source=lnt&tbs=cdr%3A1%2Ccd_min%3A1%2F1%2F2020%2Ccd_max%3A31%2F12%2F2020&tbm=nws**](https://www.google.com/search?q=Kerala+Rare+disease+policy+india&lr=&safe=images&biw=1440&bih=789&sxsrf=ALiCzsZGq96U-NugrG95kBtzLr6Q-0ovhg%3A1667724042701&source=lnt&tbs=cdr%3A1%2Ccd_min%3A1%2F1%2F2020%2Ccd_max%3A31%2F12%2F2020&tbm=nws)

**Remarks: `1 relevant article found from 5 pages**

1. [**https://www.aninews.in/news/business/dr-lal-pathlabs-ltd-collaborates-with-ordi-to-sensitize-india-on-rare-diseases20190305161730/**](https://www.aninews.in/news/business/dr-lal-pathlabs-ltd-collaborates-with-ordi-to-sensitize-india-on-rare-diseases20190305161730/) **- No relevant quotes**

**CODING DONE**

**2021**

1. **Keyword - MOHFW Rare disease policy India; Year - 1 Jan 2021 to 31 Dec 2021**

**Link :** [**https://www.google.com/search?q=MOHFW+Rare+disease+policy+india&lr=&safe=images&biw=1440&bih=789&sxsrf=ALiCzsa50hvPmKRwKOUXpawy2QMeiANwxA%3A1667724186905&source=lnt&tbs=cdr%3A1%2Ccd_min%3A1%2F1%2F2021%2Ccd_max%3A12%2F31%2F2021&tbm=nws**](https://www.google.com/search?q=MOHFW+Rare+disease+policy+india&lr=&safe=images&biw=1440&bih=789&sxsrf=ALiCzsa50hvPmKRwKOUXpawy2QMeiANwxA%3A1667724186905&source=lnt&tbs=cdr%3A1%2Ccd_min%3A1%2F1%2F2021%2Ccd_max%3A12%2F31%2F2021&tbm=nws)

1. [**https://ehealth.eletsonline.com/2021/08/govt-launches-national-portal-for-persons-living-with-rare-diseases/**](https://ehealth.eletsonline.com/2021/08/govt-launches-national-portal-for-persons-living-with-rare-diseases/) **- No quotes**
2. [**https://www.indiatvnews.com/health/know-what-diseases-fall-under-rare-diseases-category-691930**](https://www.indiatvnews.com/health/know-what-diseases-fall-under-rare-diseases-category-691930)
3. [**https://www.india.com/health/rare-diseases-all-about-treatment-challenges-and-why-should-india-take-them-seriously-4547527/**](https://www.india.com/health/rare-diseases-all-about-treatment-challenges-and-why-should-india-take-them-seriously-4547527/)

**Remarks : 3 relevant articles found**

1. **Keyword - Niti Aayog Rare disease policy India; Year -1 Jan 2021 to 31 Dec 2021**

**Link :**

[**https://www.google.com/search?q=Niti+aayog+Rare+disease+policy+india&lr=&safe=images&biw=1440&bih=789&sxsrf=ALiCzsaPH9DbgYUGVBlzq-40JhOD10mbJQ%3A1667724628523&source=lnt&tbs=cdr%3A1%2Ccd_min%3A1%2F1%2F2021%2Ccd_max%3A12%2F31%2F2021&tbm=nws**](https://www.google.com/search?q=Niti+aayog+Rare+disease+policy+india&lr=&safe=images&biw=1440&bih=789&sxsrf=ALiCzsaPH9DbgYUGVBlzq-40JhOD10mbJQ%3A1667724628523&source=lnt&tbs=cdr%3A1%2Ccd_min%3A1%2F1%2F2021%2Ccd_max%3A12%2F31%2F2021&tbm=nws)

**Remarks : No relevant articles found**

1. **Keyword - Ministry of finance Rare disease policy India; Year - 1 Jan 2021 to 31 Dec 2021**

**Link :**

[**https://www.google.com/search?q=Niti+aayog+Rare+disease+policy+india&lr=&safe=images&biw=1440&bih=789&sxsrf=ALiCzsY832VGoKNBjLU9MT4GO79EgiCILQ%3A1667723081495&source=lnt&tbs=cdr%3A1%2Ccd_min%3A1%2F1%2F2020%2Ccd_max%3A12%2F31%2F2020&tbm=nws**](https://www.google.com/search?q=Niti+aayog+Rare+disease+policy+india&lr=&safe=images&biw=1440&bih=789&sxsrf=ALiCzsY832VGoKNBjLU9MT4GO79EgiCILQ%3A1667723081495&source=lnt&tbs=cdr%3A1%2Ccd_min%3A1%2F1%2F2020%2Ccd_max%3A12%2F31%2F2020&tbm=nws)

1. [**https://health.economictimes.indiatimes.com/news/finance/govt-proposes-to-establish-alternate-funding-increase-assistance-in-new-rare-disease-policy-draft/81828869**](https://health.economictimes.indiatimes.com/news/finance/govt-proposes-to-establish-alternate-funding-increase-assistance-in-new-rare-disease-policy-draft/81828869) **- Unable to copy paste**

**Remarks: 1 relevant articles found from 5 pages**

**4. Keyword - CDSCO Rare disease policy India; Year - 1 Jan 2021 to 31 Dec 2021**

**Link:** [**https://www.google.com/search?q=CDSCO+Rare+disease+policy+india&lr=&safe=images&biw=1440&bih=789&sxsrf=ALiCzsaXymuV4r7TCDxbctPkl7tGfrjVNw%3A1667723369669&source=lnt&tbs=cdr%3A1%2Ccd_min%3A1%2F1%2F2020%2Ccd_max%3A12%2F31%2F2020&tbm=nws**](https://www.google.com/search?q=CDSCO+Rare+disease+policy+india&lr=&safe=images&biw=1440&bih=789&sxsrf=ALiCzsaXymuV4r7TCDxbctPkl7tGfrjVNw%3A1667723369669&source=lnt&tbs=cdr%3A1%2Ccd_min%3A1%2F1%2F2020%2Ccd_max%3A12%2F31%2F2020&tbm=nws)

**Remarks : No relevant articles found**

**5. Keyword - DCGI Rare disease policy India; Year -1 Jan 2021 to 31 Dec 2021**

**Link:**

[**https://www.google.com/search?q=DCGI+Rare+disease+policy+india&lr=&safe=images&biw=1440&bih=789&sxsrf=ALiCzsYwhmqnHxfsqfCJro0ip86n3WESUA%3A1667026332879&source=lnt&tbs=cdr%3A1%2Ccd_min%3A1%2F1%2F2019%2Ccd_max%3A12%2F31%2F2019&tbm=nws**](https://www.google.com/search?q=DCGI+Rare+disease+policy+india&lr=&safe=images&biw=1440&bih=789&sxsrf=ALiCzsYwhmqnHxfsqfCJro0ip86n3WESUA%3A1667026332879&source=lnt&tbs=cdr%3A1%2Ccd_min%3A1%2F1%2F2019%2Ccd_max%3A12%2F31%2F2019&tbm=nws)

**Remarks : No relevant articles found**

**6. Keyword - Court Rare disease policy India; Year - 1 Jan 2021 to 31 Dec 2021**

**Link:**

[**https://www.google.com/search?q=court+Rare+disease+policy+india&lr=&safe=images&biw=1440&bih=789&sxsrf=ALiCzsZ8jqlp-t7Qw508RKOmVb5w2xFN6Q%3A1667725420442&source=lnt&tbs=cdr%3A1%2Ccd_min%3A1%2F1%2F2021%2Ccd_max%3A12%2F31%2F2021&tbm=nws**](https://www.google.com/search?q=court+Rare+disease+policy+india&lr=&safe=images&biw=1440&bih=789&sxsrf=ALiCzsZ8jqlp-t7Qw508RKOmVb5w2xFN6Q%3A1667725420442&source=lnt&tbs=cdr%3A1%2Ccd_min%3A1%2F1%2F2021%2Ccd_max%3A12%2F31%2F2021&tbm=nws)

**Remarks : 1 relevant article found**

1. [**https://www.orfonline.org/expert-speak/rare-diseases-in-india-still-a-blind-spot/**](https://www.orfonline.org/expert-speak/rare-diseases-in-india-still-a-blind-spot/)
2. [**https://www.livelaw.in/news-updates/delhi-high-court-orders-treatment-children-suffering-rare-diseases-directs-centre-ensure-availability-necessary-funds-187636**](https://www.livelaw.in/news-updates/delhi-high-court-orders-treatment-children-suffering-rare-diseases-directs-centre-ensure-availability-necessary-funds-187636) **- need subscription**
3. [**https://www.thehindu.com/news/cities/Delhi/hc-asks-govt-aiims-to-facilitate-free-treatment-of-children-with-rare-diseases/article33777103.ece**](https://www.thehindu.com/news/cities/Delhi/hc-asks-govt-aiims-to-facilitate-free-treatment-of-children-with-rare-diseases/article33777103.ece)
4. [**https://www.hindustantimes.com/cities/others/kerala-hc-orders-setting-up-of-medical-board-to-examine-child-with-rare-disease-101625640318742.html**](https://www.hindustantimes.com/cities/others/kerala-hc-orders-setting-up-of-medical-board-to-examine-child-with-rare-disease-101625640318742.html)

**7. Keyword - Karnataka Rare disease policy India; Year - 1 Jan 2021 to 31 Dec 2021**

**Link:**

[**https://www.google.com/search?q=Karnataka+Rare+disease+policy+india&lr=&safe=images&biw=1440&bih=789&sxsrf=ALiCzsaEK1c6KgpBPZbXUPKjMISgEf3JFA%3A1667725401164&source=lnt&tbs=cdr%3A1%2Ccd_min%3A1%2F1%2F2021%2Ccd_max%3A12%2F31%2F2021&tbm=nws**](https://www.google.com/search?q=Karnataka+Rare+disease+policy+india&lr=&safe=images&biw=1440&bih=789&sxsrf=ALiCzsaEK1c6KgpBPZbXUPKjMISgEf3JFA%3A1667725401164&source=lnt&tbs=cdr%3A1%2Ccd_min%3A1%2F1%2F2021%2Ccd_max%3A12%2F31%2F2021&tbm=nws)

**Remarks: No relevant article found**

**8. Keyword - Kerala Rare disease policy India; Year -1 Jan 2021 to 31 Dec 2021**

**Link:**

[**https://www.google.com/search?q=Kerala+Rare+disease+policy+india&lr=&safe=images&biw=1440&bih=789&sxsrf=ALiCzsZO9AeLBCoIvj2SRUkYhnq-5efDOw%3A1667725541559&source=lnt&tbs=cdr%3A1%2Ccd_min%3A1%2F1%2F2021%2Ccd_max%3A12%2F31%2F2021&tbm=nws**](https://www.google.com/search?q=Kerala+Rare+disease+policy+india&lr=&safe=images&biw=1440&bih=789&sxsrf=ALiCzsZO9AeLBCoIvj2SRUkYhnq-5efDOw%3A1667725541559&source=lnt&tbs=cdr%3A1%2Ccd_min%3A1%2F1%2F2021%2Ccd_max%3A12%2F31%2F2021&tbm=nws)

**Remarks: No relevant article found**

**2022**

1. **Keyword - MOHFW Rare disease policy India; Year - 1 Jan 2022 to 30 Sep 2022**

**Link :** [**https://www.google.com/search?q=MOHFW+Rare+disease+policy+india&lr=&safe=images&biw=1440&bih=789&sxsrf=ALiCzsZYa3apaRzzOfYvvWO8o-AwrBtNog%3A1667742047666&source=lnt&tbs=cdr%3A1%2Ccd_min%3A1%2F1%2F2022%2Ccd_max%3A9%2F30%2F2022&tbm=nws**](https://www.google.com/search?q=MOHFW+Rare+disease+policy+india&lr=&safe=images&biw=1440&bih=789&sxsrf=ALiCzsZYa3apaRzzOfYvvWO8o-AwrBtNog%3A1667742047666&source=lnt&tbs=cdr%3A1%2Ccd_min%3A1%2F1%2F2022%2Ccd_max%3A9%2F30%2F2022&tbm=nws)

1. [**https://medicaldialogues.in/news/health/government-policies/50-lack-financial-aid-for-rare-disease-patients-union-health-ministry-93328**](https://medicaldialogues.in/news/health/government-policies/50-lack-financial-aid-for-rare-disease-patients-union-health-ministry-93328) **- Need subscription**

**Remarks : 3 relevant articles found**

1. **Keyword - Niti Aayog Rare disease policy India; Year - 1 Jan 2022 to 30 Sep 2022**

**Link :**

[**https://www.google.com/search?q=Niti+Aayog+Rare+disease+policy+India&lr=&safe=images&biw=1280&bih=689&tbs=cdr%3A1%2Ccd_min%3A1%2F1%2F2022%2Ccd_max%3A9%2F30%2F2022&tbm=nws&sxsrf=ALiCzsZn68NIrjGq92lNn6XOVYlZqbFTXw%3A1667742086037&ei=hrlnY7LaAcDQseMPyLmykAc&ved=0ahUKEwiytfW015n7AhVAaGwGHcicDHI4ChDh1QMIDQ&uact=5&oq=Niti+Aayog+Rare+disease+policy+India&gs_lp=Egxnd3Mtd2l6LW5ld3O4AQP4AQL4AQEyBRAAGKIEMgUQABiiBMICBxAAGB4YogRItRJQ_wNY_wNwAHgAyAEAkAEAmAGvAaAB3AKqAQMwLjKIBgE&sclient=gws-wiz-news**](https://www.google.com/search?q=Niti+Aayog+Rare+disease+policy+India&lr=&safe=images&biw=1280&bih=689&tbs=cdr%3A1%2Ccd_min%3A1%2F1%2F2022%2Ccd_max%3A9%2F30%2F2022&tbm=nws&sxsrf=ALiCzsZn68NIrjGq92lNn6XOVYlZqbFTXw%3A1667742086037&ei=hrlnY7LaAcDQseMPyLmykAc&ved=0ahUKEwiytfW015n7AhVAaGwGHcicDHI4ChDh1QMIDQ&uact=5&oq=Niti+Aayog+Rare+disease+policy+India&gs_lp=Egxnd3Mtd2l6LW5ld3O4AQP4AQL4AQEyBRAAGKIEMgUQABiiBMICBxAAGB4YogRItRJQ_wNY_wNwAHgAyAEAkAEAmAGvAaAB3AKqAQMwLjKIBgE&sclient=gws-wiz-news)

**Remarks : No relevant articles found**

1. **Keyword - Ministry of finance Rare disease policy India; Year - 1 Jan 2022 to 30 Sep 2022**

**Link :**

[**https://www.google.com/search?q=+Ministry+of+finance+Rare+disease+policy+India&lr=&safe=images&biw=1280&bih=689&tbs=cdr%3A1%2Ccd_min%3A1%2F1%2F2022%2Ccd_max%3A9%2F30%2F2022&tbm=nws&sxsrf=ALiCzsaXqUb_DsH8gGdiSJn3gR_l8lXUEA%3A1667742237167&ei=HbpnY6DyCeTjseMP9am70AM&ved=0ahUKEwjg9P3815n7AhXkcWwGHfXUDjoQ4dUDCA0&uact=5&oq=+Ministry+of+finance+Rare+disease+policy+India&gs_lp=Egxnd3Mtd2l6LW5ld3O4AQP4AQL4AQEyCBAhGBYYHhgdSNQIUABYAHAAeADIAQCQAQCYAbQBoAG0AaoBAzAuMYgGAQ&sclient=gws-wiz-news**](https://www.google.com/search?q=+Ministry+of+finance+Rare+disease+policy+India&lr=&safe=images&biw=1280&bih=689&tbs=cdr%3A1%2Ccd_min%3A1%2F1%2F2022%2Ccd_max%3A9%2F30%2F2022&tbm=nws&sxsrf=ALiCzsaXqUb_DsH8gGdiSJn3gR_l8lXUEA%3A1667742237167&ei=HbpnY6DyCeTjseMP9am70AM&ved=0ahUKEwjg9P3815n7AhXkcWwGHfXUDjoQ4dUDCA0&uact=5&oq=+Ministry+of+finance+Rare+disease+policy+India&gs_lp=Egxnd3Mtd2l6LW5ld3O4AQP4AQL4AQEyCBAhGBYYHhgdSNQIUABYAHAAeADIAQCQAQCYAbQBoAG0AaoBAzAuMYgGAQ&sclient=gws-wiz-news)

1. [**https://www.financialexpress.com/healthcare/why-a-national-policy-on-rare-diseases-banking-solely-on-non-state-actors-wont-save-lives/2446836/**](https://www.financialexpress.com/healthcare/why-a-national-policy-on-rare-diseases-banking-solely-on-non-state-actors-wont-save-lives/2446836/)
2. [**https://www.hindustantimes.com/cities/mumbai-news/rare-disease-india-foundation-writes-to-centre-for-funds-101646075206209.html**](https://www.hindustantimes.com/cities/mumbai-news/rare-disease-india-foundation-writes-to-centre-for-funds-101646075206209.html) **- no relevant quotes**

**Remarks: 2 relevant articles found**

**4. Keyword - CDSCO Rare disease policy India; Year - 1 Jan 2022 to 30 Sep 2022**

**Link:** [**https://www.google.com/search?q=CDSCO+Rare+disease+policy+India&lr=&safe=images&biw=1280&bih=689&tbs=cdr%3A1%2Ccd_min%3A1%2F1%2F2022%2Ccd_max%3A9%2F30%2F2022&tbm=nws&sxsrf=ALiCzsYi6pZX2N7_2bUV521NvtAVKACoIQ%3A1667742287452&ei=T7pnY8GYG5KUseMPgcaM8AM&ved=0ahUKEwiB_PqU2Jn7AhUSSmwGHQEjAz4Q4dUDCA0&uact=5&oq=CDSCO+Rare+disease+policy+India&gs_lp=Egxnd3Mtd2l6LW5ld3O4AQNIAFAAWABwAHgAyAEAkAEAmAEAoAEAqgEA&sclient=gws-wiz-news**](https://www.google.com/search?q=CDSCO+Rare+disease+policy+India&lr=&safe=images&biw=1280&bih=689&tbs=cdr%3A1%2Ccd_min%3A1%2F1%2F2022%2Ccd_max%3A9%2F30%2F2022&tbm=nws&sxsrf=ALiCzsYi6pZX2N7_2bUV521NvtAVKACoIQ%3A1667742287452&ei=T7pnY8GYG5KUseMPgcaM8AM&ved=0ahUKEwiB_PqU2Jn7AhUSSmwGHQEjAz4Q4dUDCA0&uact=5&oq=CDSCO+Rare+disease+policy+India&gs_lp=Egxnd3Mtd2l6LW5ld3O4AQNIAFAAWABwAHgAyAEAkAEAmAEAoAEAqgEA&sclient=gws-wiz-news)

**Remarks : 1 relevant article found**

1. [**https://www.lexology.com/library/detail.aspx?g=8c24664d-4f3f-47a1-8c86-41a972b772fb**](https://www.lexology.com/library/detail.aspx?g=8c24664d-4f3f-47a1-8c86-41a972b772fb) **- No quotes**

**5. Keyword - DCGI Rare disease policy India; Year 1 Jan 2022 to 30 Sep 2022**

**Link:**

[**https://www.google.com/search?q=DCGI+Rare+disease+policy+India&lr=&safe=images&biw=1280&bih=689&tbs=cdr%3A1%2Ccd_min%3A1%2F1%2F2022%2Ccd_max%3A9%2F30%2F2022&tbm=nws&sxsrf=ALiCzsbT1d-3Gd9NsDBj-z2fjjlV7Cz3tA%3A1667742783867&ei=P7xnY_jKNP2cseMPxbKB0AY&ved=0ahUKEwi45tWB2pn7AhV9TmwGHUVZAGoQ4dUDCA0&uact=5&oq=DCGI+Rare+disease+policy+India&gs_lp=Egxnd3Mtd2l6LW5ld3O4AQP4AQL4AQEyBRAAGKIEMgUQABiiBDIFEAAYogRIkQdQAFgAcAB4AMgBAJABAJgBkAGgAZABqgEDMC4xiAYB&sclient=gws-wiz-news**](https://www.google.com/search?q=DCGI+Rare+disease+policy+India&lr=&safe=images&biw=1280&bih=689&tbs=cdr%3A1%2Ccd_min%3A1%2F1%2F2022%2Ccd_max%3A9%2F30%2F2022&tbm=nws&sxsrf=ALiCzsbT1d-3Gd9NsDBj-z2fjjlV7Cz3tA%3A1667742783867&ei=P7xnY_jKNP2cseMPxbKB0AY&ved=0ahUKEwi45tWB2pn7AhV9TmwGHUVZAGoQ4dUDCA0&uact=5&oq=DCGI+Rare+disease+policy+India&gs_lp=Egxnd3Mtd2l6LW5ld3O4AQP4AQL4AQEyBRAAGKIEMgUQABiiBDIFEAAYogRIkQdQAFgAcAB4AMgBAJABAJgBkAGgAZABqgEDMC4xiAYB&sclient=gws-wiz-news)

1. [**https://timesofindia.indiatimes.com/blogs/voices/need-for-urgency-in-treatment-for-rare-diseases/**](https://timesofindia.indiatimes.com/blogs/voices/need-for-urgency-in-treatment-for-rare-diseases/) **- No quotes**

**Remarks : 1 relevant article found**

**6. Keyword - Court Rare disease policy India; Year - 1 Jan 2022 to 30 Sep 2022**

**Link:**

[**https://www.google.com/search?q=Court+Rare+disease+policy+India&lr=&safe=images&tbs=cdr:1,cd_min:1/1/2022,cd_max:9/30/2022&tbm=nws&sxsrf=ALiCzsbnjoRLvkUP-0qy5s7oGebrX5HSCA:1667743018859&ei=Kr1nY8KLNM6NseMPsoCzqAQ&start=10&sa=N&ved=2ahUKEwjCyNzx2pn7AhXORmwGHTLADEUQ8tMDegQIARAE&biw=1280&bih=689&dpr=1**](https://www.google.com/search?q=Court+Rare+disease+policy+India&lr=&safe=images&tbs=cdr:1,cd_min:1/1/2022,cd_max:9/30/2022&tbm=nws&sxsrf=ALiCzsbnjoRLvkUP-0qy5s7oGebrX5HSCA:1667743018859&ei=Kr1nY8KLNM6NseMPsoCzqAQ&start=10&sa=N&ved=2ahUKEwjCyNzx2pn7AhXORmwGHTLADEUQ8tMDegQIARAE&biw=1280&bih=689&dpr=1)

**Remarks : 1 relevant article found**

1. [**https://www.hindustantimes.com/opinion/rare-diseases-small-numbers-huge-burden-101645771957231.html**](https://www.hindustantimes.com/opinion/rare-diseases-small-numbers-huge-burden-101645771957231.html) **- No quotes**
2. [**https://www.livelaw.in/news-updates/can-financial-aid-rare-diseases-treatment-expenses-clinical-trials-experimental-dmd-drug-delhi-high-court-centre-206150**](https://www.livelaw.in/news-updates/can-financial-aid-rare-diseases-treatment-expenses-clinical-trials-experimental-dmd-drug-delhi-high-court-centre-206150) **- need subscription**

**7. Keyword - Karnataka Rare disease policy India; Year - 1 Jan 2022 to 30 Sep 2022**

**Link:**

[**https://www.google.com/search?q=Karnataka+Rare+disease+policy+India&lr=&safe=images&tbs=cdr:1,cd_min:1/1/2022,cd_max:9/30/2022&tbm=nws&sxsrf=ALiCzsaWwPtrBf-QGb5j-IknE-V_JU2Arg:1667743195074&ei=271nY66RBIufseMP1cqQmAM&start=0&sa=N&ved=2ahUKEwju6t_F25n7AhWLT2wGHVUlBDM4ChDy0wN6BAgBEAQ&biw=1280&bih=689&dpr=1**](https://www.google.com/search?q=Karnataka+Rare+disease+policy+India&lr=&safe=images&tbs=cdr:1,cd_min:1/1/2022,cd_max:9/30/2022&tbm=nws&sxsrf=ALiCzsaWwPtrBf-QGb5j-IknE-V_JU2Arg:1667743195074&ei=271nY66RBIufseMP1cqQmAM&start=0&sa=N&ved=2ahUKEwju6t_F25n7AhWLT2wGHVUlBDM4ChDy0wN6BAgBEAQ&biw=1280&bih=689&dpr=1)

**Remarks: No relevant article found**

1. [**https://www.expresshealthcare.in/news/karnataka-launches-new-research-centre-for-rare-diseases/435919/**](https://www.expresshealthcare.in/news/karnataka-launches-new-research-centre-for-rare-diseases/435919/)
2. [**https://www.livelaw.in/news-updates/karnataka-high-court-rare-diseases-treatment-policies-implementation-204401**](https://www.livelaw.in/news-updates/karnataka-high-court-rare-diseases-treatment-policies-implementation-204401) **- need subscription**
3. [**https://thelogicalindian.com/good-governance/karnataka-govt-to-cap-prices-of-high-cost-treatments-for-scst-communities-37328**](https://thelogicalindian.com/good-governance/karnataka-govt-to-cap-prices-of-high-cost-treatments-for-scst-communities-37328) **- no quotes**

**8. Keyword - Kerala Rare disease policy India; Year 1 Jan 2022 to 30 Sep 2022**

**Link:**

[**https://www.google.com/search?q=+Kerala+Rare+disease+policy+India&lr=&safe=images&biw=1280&bih=689&tbs=cdr%3A1%2Ccd_min%3A1%2F1%2F2022%2Ccd_max%3A9%2F30%2F2022&tbm=nws&sxsrf=ALiCzsZSo6QsLjMQpyomzMspdRkS8i6H-Q%3A1667743345519&ei=cb5nY_GbH76aseMPl4W34A0&ved=0ahUKEwixmL6N3Jn7AhU-TWwGHZfCDdwQ4dUDCA0&uact=5&oq=+Kerala+Rare+disease+policy+India&gs_lp=Egxnd3Mtd2l6LW5ld3O4AQP4AQL4AQEyCBAhGBYYHhgdMggQIRgWGB4YHTIIECEYFhgeGB1IqQdQAFgAcAB4AMgBAJABAJgBiQGgAYkBqgEDMC4xiAYB&sclient=gws-wiz-news**](https://www.google.com/search?q=+Kerala+Rare+disease+policy+India&lr=&safe=images&biw=1280&bih=689&tbs=cdr%3A1%2Ccd_min%3A1%2F1%2F2022%2Ccd_max%3A9%2F30%2F2022&tbm=nws&sxsrf=ALiCzsZSo6QsLjMQpyomzMspdRkS8i6H-Q%3A1667743345519&ei=cb5nY_GbH76aseMPl4W34A0&ved=0ahUKEwixmL6N3Jn7AhU-TWwGHZfCDdwQ4dUDCA0&uact=5&oq=+Kerala+Rare+disease+policy+India&gs_lp=Egxnd3Mtd2l6LW5ld3O4AQP4AQL4AQEyCBAhGBYYHhgdMggQIRgWGB4YHTIIECEYFhgeGB1IqQdQAFgAcAB4AMgBAJABAJgBiQGgAYkBqgEDMC4xiAYB&sclient=gws-wiz-news)

**Multinational Companies**

**Keyword - *Company Name* Rare disease India; Year -1 Jan 2021 to 30 Sep 2022**

[**https://www.thehindubusinessline.com/companies/takeda-brings-drugs-for-3-rare-diseases-to-india-but-pricing-concerns-remain/article28447477.ece**](https://www.thehindubusinessline.com/companies/takeda-brings-drugs-for-3-rare-diseases-to-india-but-pricing-concerns-remain/article28447477.ece) **(July 15 2019)**

[**https://www.healthcareradius.in/features/29955-takeda-india-launches-diagnostic-program-to-support-rare-disease-patients (30**](https://www.healthcareradius.in/features/29955-takeda-india-launches-diagnostic-program-to-support-rare-disease-patients%20%20%20(30) **June 2021)**

[**https://www.healthcareradius.in/clinical/26532-takeda-strengthens-patient-offering-in-india-via-availability-of-vedolizumab (12**](https://www.healthcareradius.in/clinical/26532-takeda-strengthens-patient-offering-in-india-via-availability-of-vedolizumab%20(12) **July 2020)**

[**https://www.biospectrumasia.com/news/25/13992/takeda-expands-india-portfolio-of-rare-disease-therapies-.html (15**](https://www.biospectrumasia.com/news/25/13992/takeda-expands-india-portfolio-of-rare-disease-therapies-.html%20(15) **July 2019)**

[**https://health.economictimes.indiatimes.com/news/pharma/takeda-launches-launches-hemophilia-drug-adynovate-in-india/91615011 (17**](https://health.economictimes.indiatimes.com/news/pharma/takeda-launches-launches-hemophilia-drug-adynovate-in-india/91615011%20(17) **May 2022)**

[**https://www.pharmaceutical-technology.com/features/improving-care-for-rare-diseases-in-india/**](https://www.pharmaceutical-technology.com/features/improving-care-for-rare-diseases-in-india/) **(5 July 2021)**

[**https://www.theguardian.com/global-development/2021/mar/15/what-price-a-childs-life-indias-quest-to-make-rare-disease-drugs-affordable** (15](https://www.theguardian.com/global-development/2021/mar/15/what-price-a-childs-life-indias-quest-to-make-rare-disease-drugs-affordable%20%20(15) **March 2021)**
