# Supplementary material for: Mapping the rare disease stakeholders in India
Source: PLOS Glob Public Health. 2026 Mar 26;6(3):e0003516. doi: 10.1371/journal.pgph.0003516 (PMC13020829; doi:10.1371/journal.pgph.0003516)
Supplement: S3 File — (DOCX) [file pgph.0003516.s003.docx]

**Additional File 1 Table of codes**

| **Categories** | **Codes** |
| --- | --- |
| 1.Experience with RD | Personal experience with RD |
|  | Professional experience with RD |
|  | Contribution to RD advocacy |
|  | Contribution to RD research |
|  | Other relevant professional experience |
|  | Contribution to other RD activities |
| 2.Perspectives on RDs and related policies | 1.Knowledge of RDs |
|  | Knowledge of disease burden in India |
|  | Knowledge of disease |
|  | 2. Knowledge of RD policies |
|  | Knowledge of Indian RD policies |
|  | Knowledge of global RD policies |
|  | 3. Treatment |
|  | Access to treatment |
|  | Medical treatment of RDs |
|  | Genetic counselling |
|  | Screening |
|  | 4. Diagnosis |
|  | Diagnostic odyssey of RDs |
|  | Importance of diagnosis |
|  | Lack of clarity in policy reg diagnosis |
|  | 5. Clinical trials |
|  | Biotech investments crucial |
|  | Facilitating easy travel and accommodation |
|  | Policy attributes on clinical trials |
|  | Providing access to clinical trials |
|  | Importance of RD clinical trials |
|  | 6. Registry |
|  | Global RD databases |
|  | Lack of access to ICMR RD registry |
|  | Need for registry and database |
|  | Pivotal role of data/ data access in RD environment |
|  | 7. Orphan drug policy |
|  | Gaps in orphan drug policy of India |
|  | Importance of global collaborations in bringing drugs |
|  | PAGs to push for local product development |
|  | Investment in R&D of orphan drugs in India |
|  | Waiver of import duty |
|  | Think tanks to advocate for orphan product manufacturers |
|  | Incentivization of orphan product manufacturers |
|  | 8. Understanding of Indian RD ecosystem |
|  | Role of PAG |
|  | Drivers for change |
|  | a. Govt policy |
|  | b. PAGs as drivers |
|  | c. Access to diagnosis |
|  | d. Networking of experts |
|  | e. Litigations by patients |
|  | f. Social media support |
|  | g. Awareness generation |
| 3. Challenges |  |
| 4. Recommendations |  |
